# Effect of Systematic Follow-Up by General Practitioners after Deliberate Self-Poisoning: A Randomised Controlled Trial

**DOI:** 10.1371/journal.pone.0143934

**Published:** 2015-12-02

**Authors:** Tine K. Grimholt, Dag Jacobsen, Ole Rikard Haavet, Leiv Sandvik, Trond Jorgensen, Astrid Berge Norheim, Oivind Ekeberg

**Affiliations:** 1 Department of Acute Medicine, Oslo University Hospital, Oslo, Norway; 2 Regional Centre of Violence, Traumatic Stress and Suicide Prevention Eastern Norway, Oslo, Norway; 3 Department of General Practice, Institute of Health and Society, University of Oslo, Oslo, Norway; 4 Department of Biostatistics, Oslo University Hospital, Oslo, Norway; 5 Psychiatric Consultation Team, Akershus University Hospital, Akershus,Norway; 6 Diakonhjemmet Hospital, Oslo,Norway; 7 Department of Behavioural Sciences in Medicine, Institute of Basic Medical Sciences, Faculty of Medicine, University of Oslo, Oslo, Norway; National Center of Neurology and Psychiatry, JAPAN

## Abstract

**Objective:**

To assess whether systematic follow-up by general practitioners (GPs) of cases of deliberate self-poisoning (DSP) by their patients decreases psychiatric symptoms and suicidal behaviour compared with current practice.

**Design:**

Randomised clinical trial with two parallel groups.

**Setting:**

General practices in Oslo and the eastern part of Akershus County.

**Participants:**

Patients aged 18–75 years admitted to hospital for DSP. We excluded patients diagnosed with psychoses, without a known GP, those not able to complete a questionnaire, and patients admitted to psychiatric in-patient care or other institutions where their GP could not follow them immediately after discharge.

**Intervention:**

The GPs received a written guideline, contacted the patients and scheduled a consultation within one week after discharge, and then provided regular consultations for six months. We randomised the patients to either intervention (n = 78) or treatment as usual (n = 98).

**Main Outcome Measures:**

Primary outcome measure was the Beck Scale for Suicide Ideation (SSI). Secondary outcomes were Beck Depression Inventory (BDI) and Beck Hopelessness Scale (BHS), self-reported further self-harm and treatment for DSP in a general hospital or an emergency medical agency (EMA). We assessed patients on entry to the trial and at three and six months. We collected data from interviews, self-report questionnaires, and hospital and EMA medical records.

**Results:**

There were no significant differences between the groups in SSI, BDI, or BHS mean scores or change from baseline to three or six months. During follow-up, self-reported DSP was 39.5% in the intervention group vs. 15.8% in controls (P = 0.009). Readmissions to general hospitals were similar (13% in both groups (P = 0.963), while DSP episodes treated at EMAs were 17% in the intervention group and 7% in the control group (P = 0.103).

**Conclusion:**

Structured follow-up by GPs after an episode of DSP had no significant effect on suicide ideation, depression or hopelessness. There was no significant difference in repeated episodes of DSP in hospitals or EMAs. However, the total number of incidents of deliberate self-harm reported by the patients was significantly higher in the intervention group.

**Trial registration:**

Trial registration ClinicalTrials.gov Identifier: NCT01342809

## Introduction

When hospitalised with deliberate self-harm (DSH), most of the admissions are because of deliberate self-poisoning (DSP) [1,2]. DSP patients often present a complex picture of mental illness and physical comorbidity due to substance abuse [3,4]. Both suicide risk and mortality from other causes are significantly increased compared with the general population [5]. Suicide attempt rates are high, especially the first year after discharge [6]. In a follow-up study after DSP, a considerable proportion of patients reported high levels of depression and need for professional help [7].

When planning aftercare, the general practitioner (GP) should have an important role [8], [9]. Also, one out of four patients reported their GP as being most important in the early discharge period [7]. Knowing the patient’s background and often also relationship to other people, a GP can maintain a broader perspective, a good continuity of care [10], and address both the DSP patient’s physical illness and psychological problems. General practice also represents a low threshold and short waiting time, which is important for this patient group reporting barriers to seeking help [11].

Randomised controlled studies have been limited in this field [12]. More evidence is required to provide sufficient aftercare and treatment choices in order to prevent further suicidal behaviour [13]. Bennewith et al. randomised patients into intervention or treatment as usual groups to follow-up after DSH [14]. The intervention comprised a letter from the GP inviting the patient to consult and guidelines to use in consultations. The attendance rate at the GP was not registered and there was no evidence that repeated DSH was lower among the patients in the intervention group. In a trial where overdose patients were randomised to either GP or outpatient clinic treatment, no significant differences were found [15]. However, low-cost universal interventions of postcard or telephone contact following DSH have shown promising results [16], [17], [18], [19,20]. Further, problem-solving therapy has given decreased depression, hopelessness and suicidal ideation among suicidal patients [21,22].

We designed a randomised controlled trial with structured follow-up by the GP after DSP. The main elements were to schedule an appointment within one week after discharge from hospital and further regular consultations for the following six months. The GPs received a guideline with suggestions of elements to address in consultations. We hypothesised that this structured follow up by GPs would decrease symptoms of suicidal ideation, depression, hopelessness and further DSH after three and six months.

## Methods

### Participants

Participants were patients admitted for DSP to acute emergency units and intensive care units in five regional hospitals in Oslo and Akershus County, Norway. We arranged meetings with the formal leaders in each hospital and provided a written trial protocol. Further, we organised cooperation with the psychiatric consultation liaison teams, physicians and nurses in the participating hospitals. We distributed envelopes with a registration form containing inclusion and exclusion criteria, written informed consent forms, the Beck Suicidal Intent Scale interview, and the baseline-questionnaire (described in the methods section). In advance, we sent an invitation to all the registered GPs with information about the trial and a written consent letter with possibility to participate or decline if a patient on their list was included in the trial. When a patient was included, we telephoned their GP to provide information about the trial and ask if they wanted to participate and follow the patient. The participating GPs then received an information letter, written consent form, the guidelines and a registration form to return after six months. For those who declined to participate, we followed the patient with questionnaires in a comparison group. The GPs in the control group received a letter with information about the patient on their list, the objectives of the trial and an invitation to treat the patient as usual and fill out a registration form.

### Eligibility criteria for participants

Eligible participants were all adults aged 18 to 75, hospitalised in acute medical wards with DSP [23], who met the eligibility criteria for receiving follow-up by their GP after discharge. We excluded patients with present psychosis, intellectual disability or organic cognitive impairment. Were also excluded patients who were not able to complete the questionnaire because of language difficulties. Patients with no GP (or unable to be registered with one, e.g. non-Norwegian citizens), those requiring transfer to psychiatric in-patient treatment or other forms of further in-patient treatment (e.g. general medical or misuse treatment) were also excluded.

### Settings

The trial was conducted at five hospitals and general practices in Oslo and Akershus County. The total period of inclusion was from November 2009 to December 2013. The area has a population of about one million.

### Intervention

The intervention group received systematic follow-up by their GP and a consultation as soon as possible, preferably within a week after discharge. Further, at least one consultation each month the first three months and then two consultations the last three months were scheduled. The GPs received short guidelines developed from the WHO guide to general practice [24] and discussed with a liaison senior psychiatrist (OE) and general practitioner (ORH). Finally, the content and its practical use were discussed and refined in a research group with GPs (S1 Fig).

The guidelines consisted of three main points:

1. To schedule an appointment as soon as possible after discharge, preferably within a week.
2. To clarify with the patient: reason for poisoning, main present problem, suicidal thoughts, details about present treatment, and need for further help.
3. Schedule further appointments.

We gave the GPs an option to contact a psychiatrist to discuss challenges during the follow-up period.

### Treatment as usual

By routine, all patients were evaluated by psychiatric personnel in the hospital before discharge. The treatment as usual consisted of referral to a psychiatric outpatient clinic, GP and other treatment modalities like family counselling or drug abuse clinics, depending on the patient’s problems and needs at the time of discharge, and during the following six months.

### Measures

We obtained data at baseline (in the hospital) and after three and six months post discharge. We interviewed the patients to fill out the Beck Suicidal Intent Scale at baseline. The patients filled out self-report questionnaires with the Beck scales of suicidal ideation, depression and hopelessness at all three times. All the Beck scales have previously been validated and translated into Norwegian.

Baseline data were demographic, clinical characteristics, and intention.

The Beck Suicidal Intent Scale (BSI) [25] is based on a clinical interview scale with 15 items referring to the patient’s precautions and beliefs about the act. The items are scored on a scale from 0 to 2, with a possible total score of 30 indicating the strongest intention of suicide. It covers precautions, planning, communication, expectations regarding the medication load, the degree of planning, and the wish to die or live.

To measure self-reported reason and intention the following options were listed: wanted to die, escape problems, influence personal relations, accident or drug related, do not remember, do not want to give reason, and other. The answer categories were yes or no. The question was based on the study by Bancroft et al. of reasons people give for taking overdoses [26].

The patients were asked whether they wanted help to solve the problems that triggered this episode (yes/no).

Demographic variables after three and six months were change or occurrence of negative life events in the patient’s life: divorce, financial problems, own or family illness, or death in close family.

### Primary outcome measure

Beck Scale for Suicide Ideation (SSI): a 19-item instrument that measures the intensity, duration and specificity of a patient’s thoughts about committing suicide. The scores range from 0–38. If the patient scores 0 on both items 4 and 5, which indicates active suicidal desire, the instruction is to skip the next 14 items, which address specific suicide plans and attitudes. The scale that measure suicide ideation (SSI) is therefore calculated for the 19 items only if the patient scores 1 or 2 on item 4 or 5, leaving a sub group with no active suicidal ideation [27]

### Secondary outcome measures

Beck Depression Inventory (BDI): this scale measures the severity of depression during the previous week. It is composed of 21 items related to depressive symptoms. Each item has a set of at least four possible answers, varying in intensity. The standard cut-offs are: scores of 0 to 9 indicate that a person is not depressed, 10 to 18 indicate mild to moderate depression, 19 to 29 moderate to severe depression and 30 to 63 severe depression [28,29]

Beck Hopelessness Scale (BHS): a 20-item scale with true/false statements for measuring positive and negative expectations about the future. The total BHS score ranges from 0 (no hopelessness) to 20 (maximum hopelessness). The classification of scores is: 0 to 3, minimal; 4 to 8, mild; 9 to 14, moderate; and 15 to 20, severe hopelessness [29].

The Cronbach’s alpha for all the Beck scales ranged from 0.74 (BDI at baseline) up to 0.93 (BDI at six months).

Repeated deliberate self-harm was measured with self-report of poisoning, cutting or other potentially harmful methods. Further, whether the patients considered the most recent episode to be a suicide attempt (yes/no).

We reviewed medical records to determine whether the general hospital or the emergency medical agencies (EMAs) treated the patients for DSP. DSP was registered in line with the same definition as used in the inclusion criteria. Contacts regarding suicide ideation without a self-inflicted overdose or methods other than poisoning were not included.

### Sample size

In a similar trial, a 5-point reduction on SSI was considered clinically significant [22]. We therefore based the sample size calculation on a mean difference of 5.0 between the intervention group and the control group. A previous trial found SD 7.69 [30]. With an alpha = 0.05 and beta = 0.2 it is necessary to include 60 patients in each group. Given an estimated dropout rate of 40%, we decided to include 200 patients in the trial.

### Randomisation

**Fig 1:**
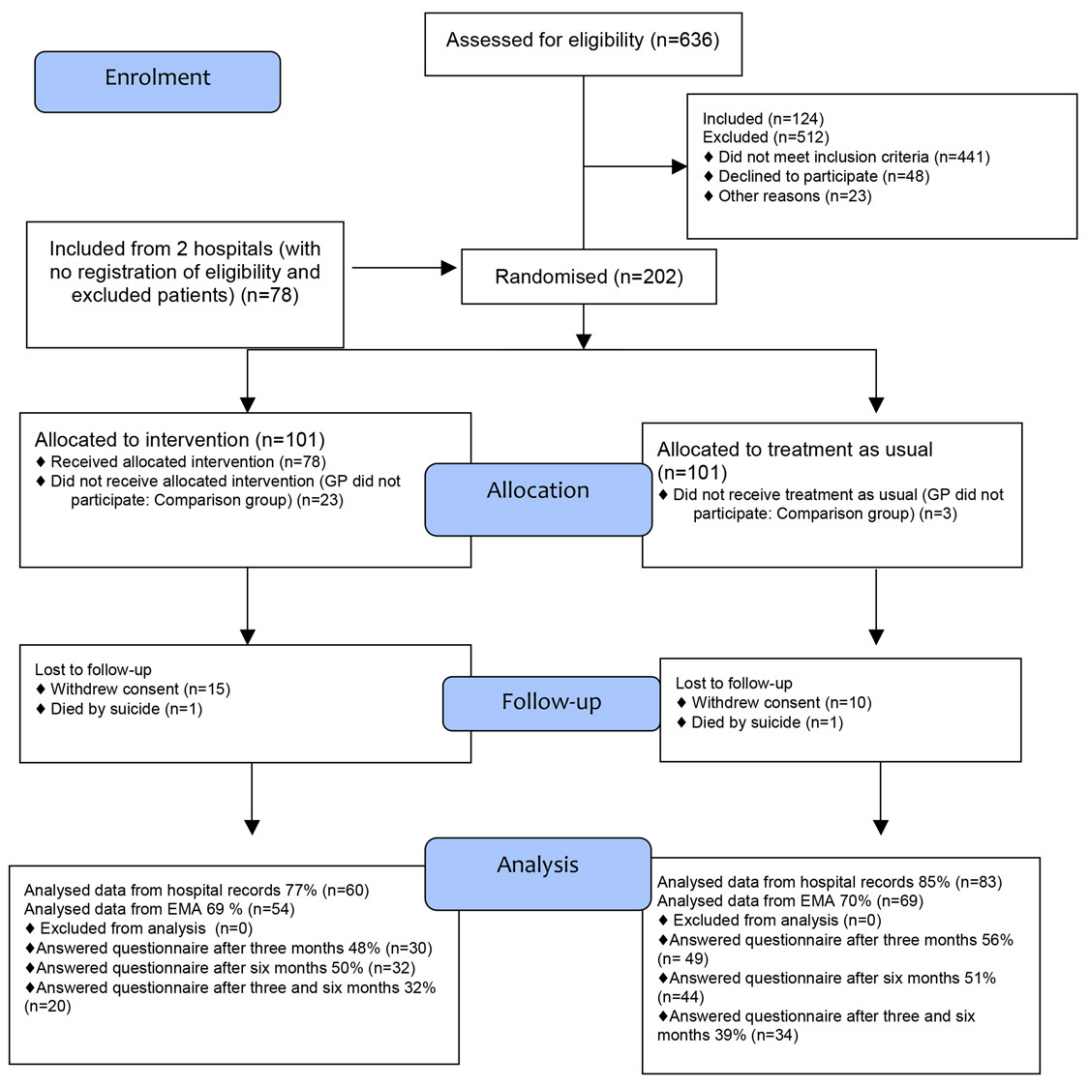
Participant flow chart.

The randomisation was 1:1 equal block distribution into the intervention or control group. Whether a patient would be allocated to the intervention or control group was determined by reference to a statistical series based on random sampling numbers. We generated a code list and randomisation plan from the web-based programme http://www.randomization.com. The list was stored at the hospital department’s secretary’s office to assure that it was not possible to manipulate it after the trial had started. We distributed envelopes containing forms and questionnaires with the code to each hospital. If a GP did not participate in the trial, either by declining beforehand or when approached, the patients were followed in a comparison group and received treatment as usual. Fig 1 shows the participant flow through the trial.

### Blinding

The patients and the assigning staff were blinded to the treatment category at the time of inclusion to prevent selection bias. The numbers were random and not predictable to assigners. The treatment as usual in the hospital was therefore not biased with regard to initiated follow-up planned at the hospital. The patients and GPs were naturally not blinded to treatment during the trial. The GPs in the control group also received a letter with information and a questionnaire about their contacts with the patient in the six-month study period. The raters were masked to allocation in more than 70% of the cases when they reviewed medical records to obtain data regarding repeated DSP in hospital and EMAs.

### Statistical methods

Means and frequencies describe demographic and clinical data. We used a chi square test to compare differences between the groups on categorical variables. We used independent sample t test for normally distributed continuous data to compare the baseline variables Cronbach’s alpha was used to calculate reliability scores for all the measures. We used mixed between-within subjects analysis of variance (ANOVA) to find differences between the intervention group and the control group on the Beck Suicide Ideation Scale, Beck Hopelessness Scale and Beck Depression Inventory.

For each missing value on a specific item, we identified the item that was most strongly associated with the selected item by using Cohen’s kappa. Then we replaced the missing value with the patient’s value on this item. If more than 50% of the values on a scale were missing for a patient, this patient’s sum-score was not included in the statistical analyses. Otherwise, missing values were imputed before the sum score was calculated. Significance level was set at P<0.05. We used SPSS (version 21.0, IBM SPSS, Armonk, NY) software to perform the analyses.

### Ethics

We informed patients at the hospital and they received written information. We obtained written informed consent from the patients, consistent with guidelines of the Oslo University Hospitals Personal Protection Agency manual and the Norwegian Ethics Committee (NEC). All the participating hospitals in the multicentre study approved the trial after the NEC. Because the assignment personnel and the patients were blinded to treatment at the time of inclusion, it was important to inform them that if they did not receive an appointment at their GP, they would receive a questionnaire by mail in three months. Further, it was important that their participation would not interfere with other planned aftercare and previous appointments. The Ethics Committee required us to telephone patients when they were sent the questionnaire to make sure that filling out sensitive questions did not trigger a suicidal crisis, and allowed just one reminder letter to be sent. We provided a telephone number in the informed consent form, and the letters at three and six months, so the patients could contact the coordinator if they had questions or wanted to leave the trial. During the trial period, we were also required by the Ethics Committee to have a back-up plan, so that if acute situations with suspected suicidal risk occurred, professional help would be offered to the patient. Because the risk of suicide and death by other causes is elevated among DSP patients, the Electronic Patient Record (Pasdoc) was used to double-check that the patient was alive before patients were mailed the questionnaire.

## Results

### Baseline data

Table 1 displays the baseline characteristics of demographic and clinical variables. There were no significant differences between the intervention and the control groups. There were 61.8% in the intervention and 51.9% in the control group who had previously been hospitalised for DSH.

**Table 1:** Demographic and clinical baseline characteristics.

	Intervention group% (n)	Control group% (n)	Test statistic	P
Sex				
Female	77.4 (48)	72.4 (63)		
Male	22.6 (14)	27.6 (24)	0.48	0.49^#^
**Mean age (95% CI)**	35.6 (32–39)	40.0 (36–43)	1.76	0.08°
**Marital status**				
Single	50.0 (30)	40.5 (34)		
Married cohabiting	43.4 (26)	41.7 (35)		
Separated/divorced	6.7 (4)	13.1 (11)		
Widowed	0.0 (0)	3.6 (3)	4.26	0.31^#^
**Living status**				
Alone	37.7 (23)	39.7 (29)		
With others	62.3 (35)	60.3 (48)	0.06	0.81^#^
**Education**				
Elementary high school	12.9 (8)	25.9 (22)		
Secondary vocational	54.8 (34)	49.4 (42)		
College/University	32.3 (20)	24.7 (21)	3.90	0.14^#^
**Employment status**				
Employed, student, military	49.2 (30)	40.5 (34)		
Unemployed	14.8 (9)	13.1 (11)		
Sick leave	6.6 (4)	11.9 (10)		
Welfare recipient	23.0 (14)	13.1 (11)		
Retired	0.0 (0)	7.1 (6)		
Maternity leave	1.6 (1)	1.2 (1)		
Other	4.9 (3)	13.1 (11)	10.57	0.10^#^
**Previously hospitalised for DSH**				
No	38.2 (21)	48.1 (37)		
Yes	61.8 (34)	51.9 (40)	1.27	0.26^#^
**Previous psychiatric outpatient treatment**				
No	44.3 (27)	45.1 (37)		
Once	11.5 (7)	17.1 (14)		
2–3 times	14.8 (9)	9.8 (8)		
4 times or more	29.5 (18)	28.0 (23)	1.51	0.68^#^
**Previous psychiatric inpatient treatment**				
No	59.0 (36)	69.9 (58)		
Once	9.8 (6)	12.0 (10)		
2–3 times	14.8 (9)	9.6 (8)		
4 times or more	16.4 (10)	8.4 (7)	3.46	0.33^#^
**Want help to solve the problems which triggered the current episode**				
No	9.8 (6)	6.2 (5)		
Yes	90.2 (55)	93.8 (76)	0.65	0.42^#^

^# ^Chi-square test.

° Students t-test.

The intention measured with the Beck Suicidal Intent Scale (BSI) was not significantly different between the groups (Table 2). Neither were there significant differences when each item in the scale was analysed separately. There were no significant differences at baseline between the intervention group and the control group on the Beck Suicide Ideation Scale, Beck Depression Inventory and Beck Hopelessness Scale. The most common reasons for DSP were to escape from problems and a wish to die (Table 2). Ten per cent in the intervention group and 6% in the control group did not want help to solve the problems that triggered the DSP episode (χ^2^ = 0.65, P = 0.42).

**Table 2:** Beck Suicidal Intent Scale, Beck Suicide Ideation Scale, Beck Depression Inventory, Beck Hopelessness Scale and Self-reported intention at baseline.

	Intervention group			Control-group				
	N	Mean	(95% CI)	N	Mean	(95% CI)	Test statistic	P
BSI	61	12.6	(11.3–13.9)	82	12.5	(10.9–14.1)	-0.10	0.92
SSI	31	16.0	(13.3–18.8)	32	17.3	(14.6–19.9)	0.65	0.52^°^
BDI	65	26.0	(23.1–29.0)	65	24.4	(21.7–27.0)	-0.85	0.4°
BHS	59	10.0	(8.7–11.4)	77	9.9	(8.7–11.2)	-0.07	0.94°
**Self-reported intention for DSP** *								
Wanted to die	62	51.5%		83	49.4%		0.07	0.79^#^
Escape problems	62	62.9%		83	56.6%		0.58	0.45^#^
Influence personal relations	62	8.1%		83	7.2%		0.04	0.85^#^
Accident or drug related	61	8.2%		84	4.8%		0.72	0.40^#^
Do not remember	61	6.6%		81	6.2%		0.01	0.92^#^
Don’t want to give reason	62	4.8%		83	3.6%		0.13	0.71^#^
Other	61	8.2%		84	13.1%		0.35	0.35^#^

*It was possible to answer in more than one category; therefore, the total exceeds 100%.

^# ^Chi-square test.

°Students t-test.

Patients lost to follow-up were not significantly different in terms of baseline demographics, clinical characteristics or the scales BSI, SSI, BDI and BHS.

### Life events during the follow-up period

A higher number of serious living problems were reported in the intervention group at three months (15.6% vs. 2.2%, (χ^2^ = 4.68, P = 0.031). In total, the prevalence of experienced negative life events after three and six months was not significantly different between the intervention and control group (41.9 vs. 46.9%, (χ^2^ = 0.19,P = 0.66) and (53.1% vs. 55.6%, χ^2^ = 0.05, P = 0.83) respectively.

### Suicide ideation, depression and hopelessness

A mixed between–within subjects analyses of variance was conducted to assess the impact of the intervention on the participants scores on the SSI, BDI and BHS across the three time periods (baseline at hospital, after three and six months follow up).

For SSI, there was no significant interaction between intervention and the time, Wilks Lambda = 0.70, *F* (2,10) = 2.08, *p = 0*.*18*, partial eta squared = 0.29.

There were no effect for time, Wilks Lambda = 0.91, *F* (2,10) = 0.15, *p* = 0.61, partial eta squared = 0. 09. In both groups the scores declined after three months, but in the intervention group it increased after six months. (Table 3). The main effect comparing the intervention and treatment as usual was not significant, *F* (1,11) = 0.15, *p* = 0.71, partial eta squared = 0.01, suggesting no effectiveness of the structured follow up from GP.

**Table 3:** Scores for the Intervention and Control groups on Beck Suicide Ideation Scale, Beck Hopelessness Inventory and Beck Depression Inventory Across three time periods.

	Intervention group			Control group			
	n	Mean	SD	n	Mean	SD	p-values
**SSI**							
Baseline	6	19.3	(5.8)	7	21.7	(7.6)	
Three months	6	17.5	(6.5)	7	21.3	(5.4)	
Six months	6	19.8	(5.3)	7	17.1	(7.2)	0.71°
							*0*.*61* °°
							*0*.*18* °°°
**BHI**							
Baseline	18	19.8	(5.8)	26	9.6	(5.9)	
Three months	18	11.2	(4.4)	26	9.6	(6.5)	
Six months	18	11.7	(5.6)	26	8.7	(6.7)	0.28°
							*0*.*78* °°
							*0*.*36* °°°
**BDI**							
Baseline	18	24.6	(8.9)	22	22.6	(10.6)	
Three months	18	22.5	(9.4)	22	21.1	(14.5)	
Six months	18	24.9	(10.1)	22	18.8	(12.1)	0.32°
							*0*.*38* °°
							*0*.*31* °°°

Between-within subjects effect analysis of variance (ANOVA).

p-values given in the table for

°Between intervention and time

°°within factors effect and

°°°interaction effect.

For BDI there were no significant interaction between intervention and time, Wilks Lambda = 0.94 *F* (2,37) = 1.20, *p* = 0.31, partial eta squared 0.06. The intervention group decreased the BDI scores after three months and increased after six months. The control group decreased the scores across all the measurement times (Table 3).

It was no substantial effect for time, Wilks Lambda = 0.95, *F* (2,37) = 1.00, *p* = 0.38, partial eta squared = 0.05. The main effect comparing the intervention and the control group was not significant, *F* (1,38) = 1.01, *p* = 0.32, partial eta squared = 0.03, suggesting no difference in the effectiveness of the structured follow up from GP.

For BHI there were no significant interaction between intervention and time, Wilks Lambda = 0.95 *F* (2,41) = 1.06, *p* = 0.36, partial eta squared 0.05. The BHS scores in the intervention group increased -, and decreased in the control group across the time period from baseline to six months (Table 3). It was no substantial effect for time, Wilks Lambda = 1.0, F (2,41) = 0.23, *p* = 0.78, partial eta squared = 0.01.

The main effect comparing systematic follow up by GP with treatment as usual was not significant, *F* (1,42) = 1.22, *p* = 0.28, partial eta squared 0.03, suggesting no difference in the effectiveness of the structured follow up from GP.

### Repeated episodes of self-harm

The self-reported DSP during follow-up was 39.5% in the intervention group vs 15.8% in the control group (χ^2^ = 6.77,P = 0.009). Cutting and other potentially harmful episodes did not differ significantly between the groups. There was no significant difference in whether the patients evaluated the episodes as suicide attempt (27.8% vs. 52.9%, (χ^2^ = 2.31, P = 0.13) (Table 4). According to the hospital medical records, 13.3% in both groups had been hospitalised for one or more repeated episodes of DSP during the six-month follow-up. The prevalence of patients treated at EMAs was 16.7% in the intervention group vs. 7.2% in the control group (χ^2^ = 2.67, P = 0.10). One patient in each group committed suicide during the follow-up.

**Table 4:** Summary results for each study group on repeated DSH after six months.

	Intervention group		Control group		Chi square test	P
	%	n (N)	%	n (N)		
Self-report from questionnaire total six-month period						
Poisoning	39.5	15 (38)	15.8	9 (57)	6.77	0.009
Cutting	25	10 (40)	15.5	9 (58)	1.36	0.24
Other self-harm	15.0	6 (40)	10.3	6 (58)	0.48	0.49
Considered episode to be suicide attempt	27.8	5 (18)	52.9	9 (17)	2.31	0.13
**Repeated DSP from review of medical records**						
Hospital* (n = 143)	13.3	8 (60)	13.3	11 (83)	0.00	0.96
Emergency medical agency# (n = 123)	16.7	9 (54)	7.2	5 (69)	2.67	0.10

*It was not possible to obtain information from medical records for all patients included because of lack of resources and reorganisation of hospitals during the inclusion period.

#Approval to view medical records was not obtained from all emergency medical agencies and so some data were omitted (n = 23).

## Discussion

### Main findings

In this randomised controlled clinical trial, structured follow-up by a GP was studied for patients presenting to a general hospital with DSP. There were no statistically significant differences between the study groups in the level of, and development of, suicide ideation, depression, or hopelessness from baseline to three or six months. Neither were there any significant differences in the number of admissions to hospitals or EMAs for DSH. However, there was significantly more DSP in the intervention group in the follow-up period without medical attention. We have no explanation why the intervention group reported more self-harm than the control group. We consider that the most reliable measure of DSP is the number of admissions to hospital, where there was no significant difference. The only statistically significant difference was on self-reported DSP, which we consider least reliable. Therefore, we think that this finding should be replicated before we make any hypotheses on possible mechanisms.

### Methodological considerations

A strength of the trial was the randomised design associated with a low risk of bias [31]. In Norway, no official surveillance of DSH exists. The diagnosis setting in hospitals according to ICD-10 (10th edition of the International Statistical Classification of Diseases and Related Health Problems) is not used systematically; hence, the distinction between acute poisoning and DSP is not reliable. A Danish trial showed that the clinical classification of a self-inflicted episode as a suicide attempt did not consistently correspond to the diagnoses in hospital records [32]. Therefore, we based the classification of repeated DSP on careful assessment of hospital and EMA records.

There was disagreement between self-reported data of DSP and hospital and EMA records. Reporting or interpretation bias may affect self-reported data, although the phrasing in the questionnaire was in line with the standardised definition. However, the patients have probably included episodes when they had not sought medical care, most likely because of less severe DSP. The finding that the patients more seldom reported the episode as a suicide attempt highlights this. A GP or psychiatric wards might also have treated episodes. From the patient’s reports, it was not possible to distinguish whether medical staff treated the episode or not.

The follow-up period of six months might have been too short. An extended follow-up period could have clarified whether the lack of effects persisted or developed further over time. One trial found that the decrease in depressive symptoms and suicidal behaviour first began at six months and continued until the end of treatment at 18 months [33]. It is, however, unlikely that the very similar pattern between the groups should differ half a year after the intervention was finished.

The repetition rate of hospitalised DSP in the current trial was 13.3%, and is about the same as the 12%–16% reported in a systematic review by Owens [34], and in studies from Denmark [32] and Baerum county, Norway [35].

However, the hospital rates from Oslo must be interpreted with caution because many patients are admitted to the EMA. This is an outpatient clinic that receives a considerable number of self-poisoning patients. Only about 17% of the severe cases are transferred to hospital, and most of the suicide attempts [36].

Seventy per cent of the eligible participants agreed to participate, and the recruitment rate was higher compared with previous studies [37,38]. The samples in this trial had similar demographic and clinical background characteristics compared with the general DSP population [39,40].

The inclusion criteria may have resulted in exclusion of people who were at somewhat higher risk of future suicidal behaviour, e.g. because those who were still suicidal after medical treatment were transferred to psychiatric wards. The withdrawal rates and missing data on the questionnaires turned out to be somewhat higher than expected despite telephone calls and reminder letters, therefore limiting the effect size and statistical power. This affects the findings because the dropouts might have responded different. And further also the generalization of the findings to a population similar to the baseline sample.

There were no significant differences between the groups in reported negative life events, but other unknown factors might have influenced the outcome. However, there were no significant differences between the groups with regard to extent, type, adherence and place of treatment received during follow-up (details will be reported separately). It is difficult, in clinical trials with a pragmatic design, to differentiate clearly the standard treatment and intervention treatment. Variations within each treatment group in the kind of treatment and amount of contact can be wide. Although several components of aftercare were registered we cannot exclude the possibility that patients in the treatment as usual group received overlapping forms of treatment or were exposed to unknown factors [41]. Missing information regarding whether the GPs actually used the guidelines further limits this knowledge of overlap.

The sample size was too small to perform sub-group analyses by age, sex, or variables related to previous DSH, psychiatric treatment or socio-economic characteristics. The scale measuring suicide ideation (SSI) is calculated for the 19 items only if the patient scores 1 or 2 on item 4 or 5. The number of patients who completed all the 19 items is therefore lower than the total. In retrospect, it is clear that the power calculation preferably should have been based on the Beck Hopelessness Inventory and Beck Depression Inventory, and the largest sample size could be chosen, so that all the outcome measures would have been fully powered. The participation in the trial per se could have represented a potential bias, because of the ethical condition of the safety procedure of telephoning all the patients after three and six months and providing an open access telephone number. There is also a possibility that the physicians in the control group may have been more careful than usual to provide better follow-up when they knew that they were participating in clinical research.

### Results in relation to other studies

Previous trials with contact following DSH have had mixed results. The missing effects in the present trial are consistent with previous studies that have also failed to show benefit on several outcomes [14,15,32,39]. Further, the finding of increased suicidal behaviour in the intervention group are consistent with a pilot trial where information regarding sources of help, telephone calls soon after presentation and a series of letters over 12 months,[39] and guidelines for GPs were provided [14]. Taken together, these negative findings of increased suicidal behaviour in follow up studies are important to highlight. Especially since the findings challenge the hypothesis that closer follow up from health care services is a key to decreased suicidal behaviour. One pitfall is that eventual previous negative results have not been published, and the importance of publishing such negative results should be stressed. One factor in clinical trials with patients presenting with suicidal behaviour is the drop out rates. It is not possible to study the patients that leave the trial, and maybe recover well. Another explanation could be that the patients that were most severely depressed chose to stay in the treatment plan and the recovered wanted to leave the trial and also the episode behind. There are many possible explanations for the negative result in the current trial, but it is not possible to draw any firm conclusions about the reasons. However, if follow up by GP increases suicidal behaviour, could it be that the amount of drug prescription also increases and give the patients easier access to an overdose? Or could a confident patient-GP relationship lower the threshold for seeking help from the GP outside office hours lead to a lower limit for taking an overdose? It is important to pay more attention also to more qualitative aspects of the follow up to gain more in depth- knowledge about such factors.

Dialectical behaviour therapy, cognitive behavioural therapy and mindfulness-based cognitive therapy have documented reduced suicidal behaviour [42,43]. Problem-solving therapy for DSH patients appears to improve depression, hopelessness, and problems [21]. These interventions are more extensive than in the present trial.

Online self-help-based Internet intervention also found improvement in suicidal thoughts and depression [44].

### Clinical implications

The levels on BDI indicated moderate to severe depression in both groups. Even though we observed a slight decline, it seems that the symptom levels are rather persistent. We found somewhat fewer symptoms in both groups, but the levels indicate that more treatment is necessary and will take time.

One study indicated that patients with a score of 9 or above on BHS are nearly 11 times more likely to die subsequently by suicide or to self-harm compared with those who score below [45]. After three and six months, the scores in the intervention group (10.6 and 9.6 respectively) also indicate the importance of more extensive treatment.

However, although the GPs might be familiar with suicide risk factors, it can be difficult to identify an impending act of suicidal behaviour, as the vast majority of the acts are impulsive [46]. Further, the majority of DSH patients are hospitalised outside office hours [47]. This might to some extent limit the GPs’ possibility for intervention.

### Further research

Although the contribution of general practice physicians has been indicated as important in suicide prevention [48], the evidence from intervention studies in general practice is sparse. Taken in conjunction with the findings from Bennewith’s trial, where more patients repeated DSH in the intervention group, it is important to confirm or reject the hypothesis that follow-up by a GP decreases suicidal behaviour. Although we added variables related to suicidal behaviour, further RCTs with higher power are needed. More tailored interventions with cooperation between GPs and e.g. psychiatric follow-up may be more relevant, in order to resolve various aspects of the intervention. In addition, interventions may be more tailored if more homogenous patient groups were studied, e.g. patients with depression, personality disorders, or other major diagnoses. It is possible that interventions that are more intensive should include education or supervision. General practitioner educational programmes were indicated as one important suicide preventive measure 20 years ago [49], and again 10 years later [50]. Norwegian GPs perceived their skills to be intermediate in taking care for suicide attempters, 39% had participated in courses and theoretical training in assessment and treatment, but most did not report a need for more training [51].

Further studies should provide sufficiently detailed information about how the intervention was actually conducted.

## Conclusion

Structured follow-up by a GP after an episode of DSP in one of their patients had no significant effect on suicide ideation, depression and hopelessness. There was no significant difference in repeated episodes of DSP in hospitals or EMAs. However, the total number of DSH episodes reported by the patients was significantly higher in the intervention group.

## Supporting Information

S1 Fig Guidelines for GPs in the intervention group. (TIF)

S1 File CONSORT 2010 Checklist. (DOC)

S2 File Ethical approval file. (PDF)

S3 File Protocol. (PDF)
