# Supplementary figures and images for: Effect of Systematic Follow-Up by General Practitioners after Deliberate Self-Poisoning: A Randomised Controlled Trial
Source: PLoS One. 2015 Dec 2;10(12):e0143934. doi: 10.1371/journal.pone.0143934 (PMC4667913; doi:10.1371/journal.pone.0143934)

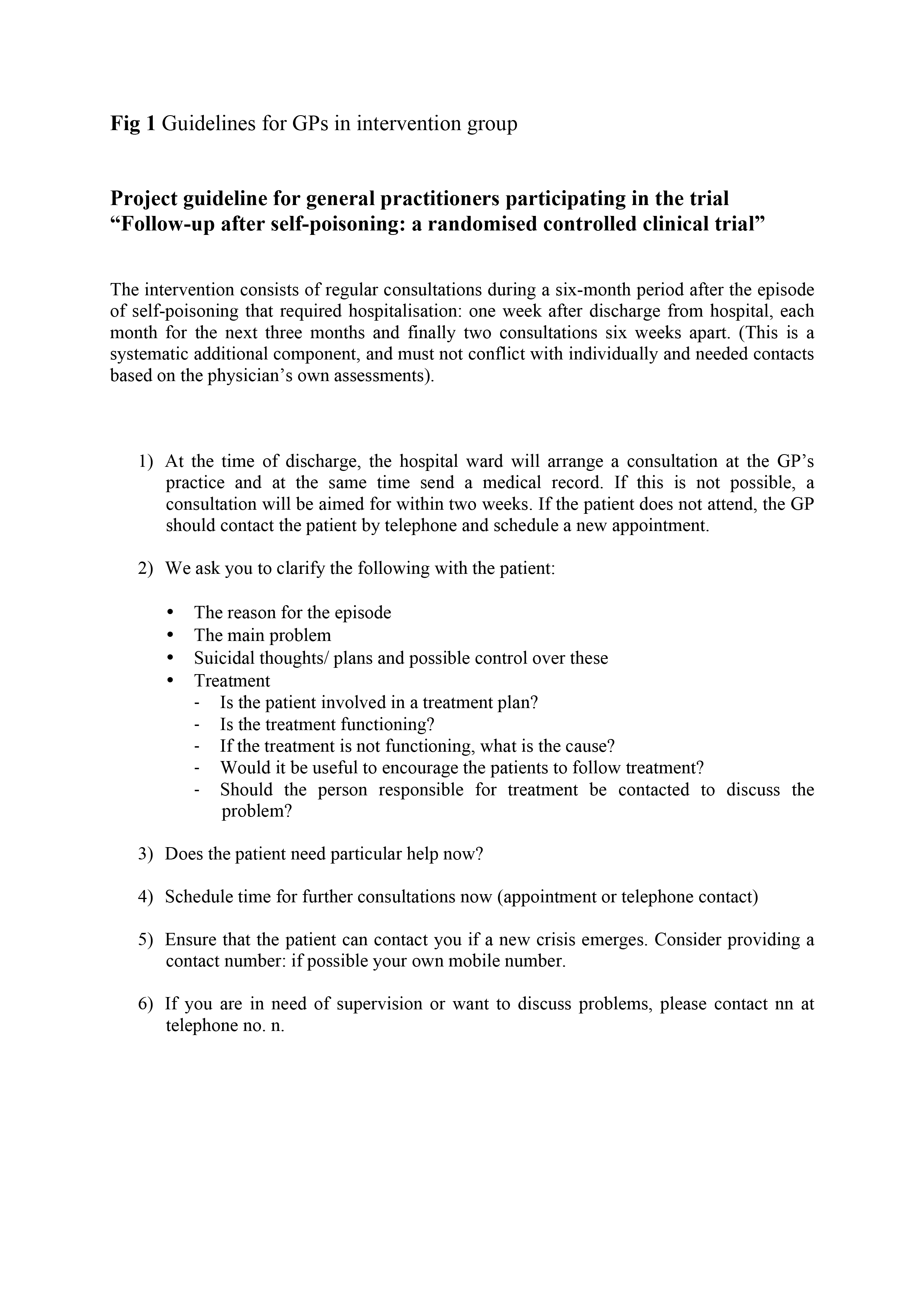

Supplement: S1 Fig — (TIF) [file pone.0143934.s001.tif]
