# Supplementary material for: Effect of Systematic Follow-Up by General Practitioners after Deliberate Self-Poisoning: A Randomised Controlled Trial
Source: PLoS One. 2015 Dec 2;10(12):e0143934. doi: 10.1371/journal.pone.0143934 (PMC4667913; doi:10.1371/journal.pone.0143934)
Supplement: S2 File — (PDF) [file pone.0143934.s003.pdf]

Skjema for etisk vurdering av forskningsprosjekter som vedrører forsøkspersoner/pasienter/klienter/informanter. (hovedskjema)

Vurderingen går til

1. Tittel

Forskningsprosjektets tittel (kortfattet tittel på norsk)

Systematisk oppfølging fra fastlegen etter et selvmordsforsøk- en randomisert kontrollert klinisk studie.

Beskriv prosjektet med 100-200 ord

Formålet med prosjektet er å: a) Studere legers holdninger og kompetanse til å møte pasienter som har vært innlagt i somatiske sykehusavdelinger etter selvmordsforsøk. b) Undersøke om mer systematisk oppfølging fra fastlegene kan redusere selvmordsatferd. c) Undersøke om fastleger som deltar i en intervensjon med oppfølging av pasienter og samtidig mottar veiledning og skriftlig undervisningsmateriale fører til endring av deres holdninger og kompetanse, den oppfølgingen de gir sine pasienter og deres tilfredshet.
Delstudie 1 er en intervensjonsstudie hvor 100 pasienter randomiseres til tettere oppfølging fra fastlegen, og 100 pasienter følges opp som ved rutinemessig behandling. Fastlegene mottar et spørreskjema og et registreringsskjema.
Delstudie 2 omfatter en spørreskjemaundersøkelse til 600 leger i Norge.

Hvis prosjektet henter materiale fra en eksisterende biobank, oppgi navn og enten prosjektnummer i REK eller registreringsnummer i Biobankregisteret

Ikke aktuelt.

2. Prosjektleder

Etternavn, fornavn	Ekeberg, Øivind
Akademisk grad/utdanning	dr.med spes. psykiatri
Stilling	Professor overlege
Arbeidssted	Akuttmedisinsk avd. Ullevål Universitetssykehus
Adresse	Kirkeveien 166
Postnummer	0407
Poststed	OSLO
Telefon	22117323
Telefaks	22117351
E-postadresse	oivind.ekeberg@uus.no

Skal denne adressen benyttes ved svar fra REK? Hvis ikke oppgi svaradresse

nei, Tinegrim@yahoo.no

Medarbeidere (navn, tittel, stilling og arbeidssted)

Tine Kristin Grimholt, sykepleier, dr.gradstipendiat / forskningssykepleier Akuttmedisinsk avdeling Ullevål US.
Dag Jacobsen, professor/ overlege, avd.leder, Akuttmedsisinsk avd. Ullevål US.
Georg Schjelderup, psykiatrisk sykepleier, teamleder selvmordsforebygging, Regionalt senter for vold traumatisk stress og selvmordsforebygging- Helse Øst

3. Prosjektbeskrivelse

Prosjektbeskrivelsen må gis i en alminnelig og forståelig språkform på norsk. Det må redegjøres kort for hensikt, hypotese, metode, tidsrom og prinsipper for utvelgelse av forsøkspersoner/informanter og aldersgrupper. Det er ikke tilstrekkelig å henvise til protokoll. Fullstendig prosjektbeskrivelse/forsøksprotokoll og evt spørreskjemaer etc. skal vedlegges.

BAKGRUNN
Fastlegene har en nøkkelfunksjon i å organisere det medisinske arbeidet rundt pasienter, kjenne deres helseproblemer og representere kontinuiteten i oppfølgingen. Når det gjelder mennesker som har forsøkt å ta sitt eget liv er det svake leddet i behandlingskjeden ofte i forbindelse med utskrivelse fra somatiske avdeling.
Forskning rundt selvmordsforebyggende tiltak, tidlig intervensjon og oppfølging har vært svært begrenset i Norge og internasjonalt.
Det er påvist at enkle former som telefonkontakt, postkort og andre former for oppsøkende kontakt virker bra for denne pasientgruppen som har avvisning som et av sine sentrale problemområder.
Tidligere studier har vist at medisinsk personale kan ha negative holdninger til selvmordsforsøkere,unngår å engasjere seg og dermed ikke fanger opp problemene pasientene sliter med. Det er ikke gjennomført studier som har kartlagt legers holdninger og kompetanse i Norge. Vi vet idag svært lite om hvilken oppfølging selvmordsforsøkere får etter at de utskrives fra somatiske avdelinger og det er lite kunnskap omkring adekvate tiltak for denne pasientgruppen.
HENSIKT
Studere fastlegers holdninger og syn på egen kompetanse til å møte og behandle pasienter med selvmordsatferd. Og sammenlikne med holdninger blant leger i indremedisinske og psykiatriske avdelinger.
Undersøke om en intervensjon som består av tettere oppfølging og koordinering av selvmordsforsøkere kan ha effekt på selvmordsatferd (selvmordstanker, repeterte forsøk), bedre oppfølging og økt pasienttilfredshet.
Undersøke om fastlegers deltakelse i en intervensjon hvor de mottar veiledning og en skriftlig manual har effekt på holdninger,syn på egen kompetanse og pasienttilfredshet.
DESIGN
Delstudie 1) Mer systematisk oppfølging fra fastleger etter selvmordsforsøk, en randomisert kontrollert klinisk studie.
Metode:
Opptaksområde
Akuttmedisinske avdelinger ved: Ullevål Universitetssykehus, Aker Universitetssykehus, Diakonhjemmet sykehus og Lovisenberg sykehus.
Inklusjonskriterier: Pasienter som innlegges i akuttmedisinske avdlinger etter selvpåførte forgiftninger med suicidal intensjon. Alder 18-75 år.
Eksklusjonskriterier: Intoksikasjoner hvor det var et åpenbart ønske om rus eller aksidentelle forgiftninger (eks. røykskader). Direkte innleggelse i psykiatrisk døgnavdeling, psykotiske og pasienter som av mentale eller språkmessige årsaker ikke kan besvare spørreskjemaer. Pasienter som bor utenfor opptaksområdet ,er under soning i fengsel eller av praktiske årsaker ikke lar seg kontakte (uten fast adresse).
Antall: Det inkluderes 200 pasienter, 120 til eksperiment- og 80 til kontrollgruppe. Pasientene i eksperimentgruppen mottar en intervensjon og kontrollgruppen "treatment as usual".
Intervensjon:
Denne består av tettere oppfølging fra fastlegen enn rutine. Pasienten innkalles til konsultasjon innen to uker etter utskrivelse fra sykehuset tilsammen tre konsultasjoner de første tre mnd og deretter to konsultasjoner de påfølgende tre måneder. Fastlegen orienterer seg om og koordinerer pasientens øvrige behandlingstilbud (DPS ol.). Fastlegen mottar skriftlig undervisningsmateriale om behandling og vurdering av pasienter med selvmordsatferd i primærhelsetjenesten. Fastlegen får i tillegg tilbud om å kontakte psykiater for generell veiledning og for å drøfte problemstillinger knyttet til sin pasient.
Kontrollgruppen mottar rutinemessig behandling som ofte er henvisning til fastlege eller oppfølging fra DPS etter utskrivelse. (Dette vil bli kartlagt)
Randomiseringsprosedyre:
Pasienter som er inkluderbare få forespørsel om deltakelse i studien i avdelingen. De mottar muntlig

og skriftlig informasjon og underskriver samtykkeerklæring. Pasientene vil kobles til en forhåndsdefinert kode som avgjør deltakelse i eksperiment eller kontrollgruppen. Både personalet og pasient er i inklusjonstidspunktet blindet for å motvirke seleksjons og gjennomføringsbias. Pasienten samtykker i å eventuelt bli kontaktet av prosjektkoordinator på telefon innen en uke etter utskrivelse ved fordeling til intervensjonsgruppe.
Inklusjonsprosedyren er todelt, slik at pasientens fastlege må informeres og samtykke til deltakelse før pasienten inviteres til intervensjonsgruppen. Listen med randomiseringskoden er generert fra et dataprogram, den vil oppbevares av personer som ikke har noen annen tilknytning til prosjektet, og det vil ikke være mulig å avsløre nummerets fordelingskode. På denne måten kan ikke ansatte i avdelingen eller prosjektledelsen påvirke fordelingen (seleksjonsbias).
Alle fastleger med pasienter på sin liste som inkluderes fra begge grupper får tilsendt spørreskjema om holdninger ved inklusjon (n= mer enn 200). (Fastleger som ikke ønsker å delta blir bedt om å fylle ut spørreskjema.)
Fastlegene i intervensjonsgruppen får i tillegg tilsendt det samme skjemaet etter seks måneder.
Det er tre måletidspunkt av pasienter i begge grupper. Etter tre og seks måneder får pasientene et spørreskjema i posten.
t1 Ved inklusjon (i sykehusavdelingen)
t2 Tre måneder etter utskrivelse
t3 Seks måneder etter utskrivelse
Bakgrunnsvariabler reg ved t1:
Kjønn, alder, tidligere psykiatrisk behandling, psykiatrisk diagnose med utgangspunkt i ICD-10.
Somatisk lidelse, tidligere selvmordsforsøk, psykiatrisk vurdering og oppfølgingstilbud ved utskrivelse.
I tillegg måles grad av selvmordsintensjon med Beck Suicide Intention Scale.
Utfallsvariabler:
t1og t2: Paykels mål på selvmordstanker, Beck scale for suicide ideation, Beck hopelessness scale og Beck Depression Inventory.
t2 og t3: Paykels mål på selvmordstanker, Beck scale for suicide ideation, Beck hopelessness scale og Beck Depression Inventory. Endringer i sosiodemografiske bakgrunnsvariabler. Nye episoder med villet egenskade med og uten innleggelse og registrering av metode.
t3: Her kommer i tillegg: Tilfredshet med fastlege måles med EUROPEP.
(Alle instrumentene er benyttet i norske studier, viser tilfredsstillende validitet og reliabilitet.
Instrumentene er ofte benyttet i det suicidologiske forskningsfeltet)
Instrumentet er validert gjennom en norsk studie.)
Registreringer fra sykehus, legevakt og fastlege:
Konsultasjoner med fastlege, telefon og brevkontakt.
Compliance med planlagt behandling ved DPS og fastlege.
Annen behandling
Delstudie 2) Kartlegging av holdninger og kompetanse blant leger
Metode:
Fastleger, leger i indremedisinske og psykiatriske avdelinger trekkes tilfeldig fra offisielle lister og får tilsendt et spørreskjema i posten.
Spørreskjemaet inneholder spørsmål om holdninger og syn på egen kompetanse for å møte og behandle pasienter med selvmordsatferd. Tilsammen 600 leger inkluderes, 200 fra hver av de tre gruppene.
Instrumenter for kartlegging av holdninger er hentet fra tilsvarende skandinaviske studier av helsepersonell.
Det sendes en purring etter 4 uker og en telefonsamtale etter seks uker.

4. Vedlegg

Presiser hvilke dokumenter som vil bli vedlagt.

1. Prosjektbeskrivelse
2. Informasjonsbrev og samtykkeerklæring til pasienter
3. Informasjonsbrev til pasienter etter tre måneder
4. Informasjonsbrev til pasienter etter seks måneder
5. Informasjonsbrev og samtykkeerklæring til fastleger i intervensjonsgruppen
6. Informasjonsbrev og samtykkeerklæring til fastleger i kontrollgruppen
7. Informasjonsbrev til leger i delstudie 2 (holdningsstudien)

8: Spørreskjema om holdninger og kompetanse til fastleger, leger i indremedisin og psykiatriske avdelinger
9: Spørreskjema til pasienter ved inklusjon
10. Spørreskjema til pasienter ved tre og seks måneder
11. Tillegg ved spørreskjema seks måneder: EUROPEP
12.Registreringsskjema for sykehusavdelingen (Inkl. Beck Suicide Intention Scale)
13. Registreringsskjema for registrering av pasienters kontakt med helsetjenester i oppfølgingsperioden.

5. Klassifisering av prosjektet

Prosjektomfang
- ○ Enkeltinstitusjon
- ◉ Nasjonal multisenterstudie
- ○ Internasjonal multisenterstudie

Prosjektet er
- ○ Studentprosjekt
- ◉ Dr.gradsprosjekt
- ○ Annet

Navn på evt. doktorand/student: Tine Kristin Grimholt

**Prosjektet er**<br>Hvis aktuelt, kryss for mer enn én	☐ Grunnforskning<br>☑ Klinisk, anvendt forksning<br>☐ Bio- og genteknologisk forskning<br>☐ Utprøving av medisinsk utstyr<br>☐ Legemiddelutprøving<br>☐ Samfunnsmed./epidemiol. forskning<br>☑ Psykologisk forskning<br>☐ Annen helsefaglig forskning<br>☐ Samfunnsvitenskapelig forskning<br>☐ Forskning i utviklingsland<br>☐ Annen
Ved annen, hva?	
Ved legemiddelutprøving, hvilken utprøvingsfase?	○ I<br>○ II<br>○ III<br>○ IV
Prosjektet er	○ Terapeutisk<br>◉ Ikke-terapeutisk

6. Prosjektomfang

Hvem inngår i studien?
Kryss av alle aktuelle

- ☐ Kun allerede registrerte data
- ☐ Friske personer
- ☑ Pasienter/syke
- ☑ Voksne
- ☐ Barn (under 18 år)
- ☐ Andre umyndige
- ☐ Kun kvinner/jenter
- ☐ Kun menn/gutter
- ☐ Innsatte i fengsel
- ☐ Soldater
- ☐ Minoritetsgrupper
- ☐ Fostre
- ☐ Lik
- ☐ Kun annet humant materiale

Ved andre umyndige, hvilke?

Ved annet humant materiale, hvilken type?

7. Inklusjon av begge kjønn

Der det er relevant, må studier dimensjoneres slik at det kan gjøres kjønnsspesifikke analyser av resultatene. Dersom ikke begge kjønn inkluderes, må dette begrunnes. Ved inklusjon av fertile kvinner skal det redegjøres for evt bruk av prevensjon og prosedyrer ved uforutsett graviditet. Ved inklusjon av gravide må prosjektleder vurdere og beskrive mulige konsekvenser for kvinnen og for fosteret og redegjøre for hvilken oppfølgning som er planlagt.

Begge kjønn inkluderes.

8. Hvor mange inngår i studien?

Antall i Norge 1000

Antall totalt 1000

Begrunn antall forsøkspersoner/informanter. Når det er relevant, gjør rede for styrkeberegning.

Delstudie 1)
Styrkeberegning:
Siden selvmord og selvmordsforsøk er relativt lavfrekvente hendelser, kreves store materialer for å vise effekt av tiltak (Gunnell & Frankel, 1994). Før det iverksettes studier med så store materialer bør det gjennomføres studier som har gitt lovende resultater. Hvis det antas at 20 % vil repetere selvmordsforsøket innen et år, vil en reduksjon til 15 % være klinisk signifikant. Med 80 % styrke og to-sidig 5 % signifikansnivå, vil det kreve ca. 900 personer i hver gruppe (Altman, 1991; Elwood,1998). Derfor legges det også vekt på endringer i selvmordsintensjon:
Beck Suicide Intenetion Scale har vist prediksjon på selvmord og selvmordsforsøk.
En reduksjon med fem poeng vil være klinisk signifikant. Basert på en tidligere studie hvor det ble funnet SD = 7,7 med alpha =0,05 og betha 0,2 er det nødvendig å inkludere 74 pasienter i hver gruppe.
Med 30 % drop out er det nødvendig å inkludere 200 pasienter.
Fastleger med en inkludert pasient på sin liste (intervensjon/ kontrollgruppe) (n= 200) får tilsendt et spørreskjema om holdninger og kompetanse.
Delstudie 2)
Det inkluderes 600 leger. Ved 50 % svarrespons vil det jfr. tilsvarende studier være tilstrekkelig antall for å utføre adekvate analyser av materialet.

9. Vitenskapelig vurdering

Studier skal gjennomføres i henhold til god vitenskapelig standard når det gjelder formål, nytte, viktighet, relevans og metode. Metodevalg, fremgangsmåte og effektmål må begrunnes.

Her gis en kort beskrivelse og det henvises til prosjektbeskrivelse for utfyllende opplysninger:
FORMÅL
Skaffe kunnskap om norske legers holdninger og kompetanse til å møte og behandle selvmordsforsøkere. Undersøke om tettere opfølging av selvmordsforsøkere reduserer selvmordsatferd i form av repeterte forsøk og selvmordstanker. Undersøke om legers holdninger og kompetanse endres i forbindelse med oppfølging av selvmordsforsøker ved å motta veiledning og en skriftlig manual. Undersøke om det er forskjell på pasienters tilfredshet med fastlegen i eksperiment og kontrollgruppe. Undersøke om det er forskjell i omfang av pasientenes behandlingstilbud og compliance med denne.
NYTTE
Kunnskap fra denne studien vil benyttes til undervisnings og kompetansehavende tiltak i samarbeid med Regionalt senter for vold, traumatisk stress og selvmordsforebygging region Øst. Dersom oppfølgingsintervensjonen viser effekt er dette en enkel og lite ressurskrevende metode å implementere i klinisk praksis. En slik form for oppfølging fra fastlegen vil i tillegg i være uavhengig av geografiske og kulturelle forskjeller.
VIKTIGHET
Forskningen omkring selvmordsforebyggende tiltak har vært svært begrenset og ikke på noen måte stått i stil til behovet for slik kunnskap. I Norge har det ikke vært gjennomført randomiserte kontrollerte studier som evaluerer effekten av enkle intervensjoner rettet mot den voksne delen av pasientgruppen. Tilsvarende studier i utlandet har også vært begrenset.
Dagens kunnskapsstatus innenfor suicidologien er høy innenfor epidemiologi og årsaksforklarende mekanismer, men har de siste 20 år stagnert når det gjelder utvikling og forskning rundt konkrete selvmordsforebyggende tiltak. Det er tillegg grunn til å huske at mennesker som forsøker å ta sitt eget liv har problemer som må tas på alvor. Selvmordsatferd er en alvorlig tilstand som rammer mange mennesker hvert år. Det er derfor ikke grunn til å rette fokus mot mortalitet og det endelige selvmord alene, men også behandle symptomer som selvmordstanker, håpløshet og depresjon. For mange av selvmordsforsøkerne kan en hjelpende hånd bidra til å få livsønsket tilbake. Enkle tiltak som å redusere årsaksutløsende forhold kan være avgjørende, eksempelvis hjelp til å rette opp økonomiske elende.
RELEVANS
Prosjektet er relevant fordi det ikke er gjort tilsvarende studier i Norge. Epidemiologiske studier viser at selvmordsatferd er et alvorlig helseproblem. Spesielt blant unge voksne er selvmord en av de vanligste dødsårsaker.
METODER
Holdningsstudien er en deskriptiv tverrsnittstudie med survey. Deltakerne er tilfeldig valgt.
I Intervensjonsstudien benyttes RCT:
Randomiserte kontrollerte studier er ansett for å være gullstandard innen vitenskap. Det er grunn til å bemerke at innenfor klinisk forskning er det umulig å forebygge alle former for bias, spesielt performance bias. Blinding er av naturlig årsaker heller ikke mulig å gjennomføre i alle ledd.
PROSJEKTGRUPPENS KOMPETANSE
Prosjektgruppen er sammensatt av personer med erfaring fra pasienter med selvmordsatferd og består av psykiatriske sykepleiere, psykiatere, psykologer. Alle deltakerne har klinisk erfaring og mange har forskererfaring. Gruppens kompetanse kan derfor iverksette adekvate tiltak for pasientene som deltar i studien dersom det er aktuelt.
For detaljert beskrivelse av prosjekt/ forskergruppen henvises til prosjektbeskrivelse.

10. Etisk vurdering

Vil prosjektet bli utført i henhold til retningslinjene i Helsinkideklarasjonen eller andre relevante etiske retningslinjer?

- (x) Ja
- ( ) Nei

Hvis disse på noe punkt ikke følges, må dette begrunnes.

Drøft etiske spørsmål som prosjektet reiser. Angi spesielt hvilke etiske betenkeligheter det er ved prosjektet og begrunn eventuelt hvorfor man kan se bort fra dem eller hvorfor man kan redusere betydningen av dem.

Etiske problemstillinger drøftes her punktvis:
1) Personvern
Personvernombudet ved Ullevål Universitetssykehus har godkjent studien. Alle prosedyrer og retningslinjer vil bli fulgt. Meldeskjema og tillatelse er vedlagt søknaden. Informasjon vil ble gitt skriftlig og muntlig. Samtykkeerklæringer er utarbeidet i samråd med personvernombudet ved UUS. Det er grunn til å merke seg følgende: Personvernombudet mente det ikke var grunn til å benytte seg av et samtykke som inneholdt muligheter for å krysse av for a) deltakelse i studien, b) fylle ut spørreskjema ved t1 og c) at vi fikk tillatelse til å bruke personnummer til å innhente opplysninger fra dødsårsaksregisteret og pasientregisteret. Vi valgte å ikke søke konsesjon om å innhente opplysninger fra disse registerne og alternativ tre utgikk. De to andre alternativene ble deretter lagt inn i teksten. Istedetfor å innhente opplysninger om dødsårsak og pasientbehandling gjennom registre valgte vi istedetfor å innhhente disse opplysningene fra sykehus, legevakt og fastlege.
Årsaken til at kolonnene ble valgt var at vi har samarbeidet med Bergljot Gjelsvik som i sin studie benyttet tilsvarende inklusjons- kriterier og setting og som hadde blitt pålagt av etisk komite å utforme samtykket på denne måten. Ved Personvernombudet ble dette imidlertid oppfattet motsatt, på grunnlag av erfaringer med behandling i etisk komite.
Det virker derfor som dette er basert på skjønn. Fordi det er ønskelig å skaffe informasjon om pasienter som ikke ønsker å delta videre i studien (frafallsanalyse) og bare besvare et spørreskjema (I Gjelsviks studie var dette 12,5 %), er dette alternativet nå fjernet, og pasienter vil få anledning til å besvare spørreskjemaet helt anonymt uten videre deltakelse. Dataene rapporteres uten direkte eller indirekte personidentifiserbare opplysninger.
2) Pasienter med redusert bevissthetstilstand:
Pasienter som innlegges i sykehus med en forgiftningstilstand har ofte redusert bevissthet. Det er viktig at pasientene er tilstrekkelig våkne, klare og orienterte før de blir forespurt om deltakelse i studien og blir bedt om å fylle ut spørreskjemaer. Personer som spør pasienter om deltakelse blir derfor instruert i å ivareta dette. Det er imidlertid grunn til å tro at dersom en pasient klarer å fylle ut et spørreskjema er tilstanden tilfredsstillende nok til å kunne ta stilling til deltakelse. Informasjonsbrevet ved inklusjon, tre og seks måneder opplyser at det er anledning til å trekke seg på et hvilket som helst tidspunkt uten å måtte oppgi noen grunn.
3) Blinding av personell i avdelingen og pasienters fordeling til intervensjon eller kontrollgruppe. Dette vil hindre systematisk skjevfordeling av pasienter.
Pasienter vil ikke falle mellom to utskrivningsopplegg dersom utskrivende lege/ psykolog ikke henviser til annen oppfølgende behandling fordi pasienten allikevel skal delta i intervensjonsgruppen og følges opp av fastlegen.
Ved at personalet i avdelingen ikke vet hvilke gruppe pasienten trekkes til, vil studiedeltakelse ikke hindre iverksetting av rutinemessige tiltak ved utskrivelse, spesielt ved sykehus der man er i ferd med å etablere rutiner for tiltak ved utskrivelse.(Dette er tilfelle ved Lovisenberg Sykehus). Det kan også forhindre at pasienten risikerer å falle mellom to behandlingsopplegg dersom han mottar redusert tiltak ved utskrivelse pga det er kjent at pasienten skal delta i intervensjonsgruppe, men mister muligheten for tettere oppfølging dersom fastlegen ikke ønsker å delta og dermed sitter igjen uten noe tilbud.
4) Hva med pasientene i kontrollgruppen, kan et avslag fra deltakelse i intervensjonsgruppen føre til økt selvmordsatferd?
For å redusere en opplevelse av avslag og ny avvisning av pasienter i kontrollgruppen er informasjonsbrevet formulert slik at intervensjonen med et oppfølgingsprogram hos fastlegen bare er tilstrekkelig beskrevet til at pasientene kan ta stilling til om de ønsker å delta og eventuellt motta en telefon innen en uke for deltakelse i intervensjonsgruppe. Pasientene som ikke får tilbud om dette har allikevel allerede fått et telefonnummer og anledning til å ta kontakt dersom de føler behov for det. (Tidligere studier hvor pasienter har fått med seg et kort med et telefonnummer og anledning til å ta kontakt har dette ført til redusert selvmordsatferd.)
5) Hva hvis fastlegen ikke ønsker å delta i intervensjonen og følge opp sine pasienter?
Det er viktig at studien ikke bidrar til å svekke et forhold mellom fastlege og pasient. Det kan være praktiske og tidsmessige årsaker annet enn et ønske om å ikke følge opp pasientene sine etter selvmorforsøk som ligger til grunn ved et avslag om deltakelse fra fastlegen. For å ivareta fastlegenes mulighet til å takke nei til deltakelse og samtidig ikke informere pasientene om deres avslag, blir

fastlegen kontaktet før pasientene (for pasienter som ikke ønsker at fastlegen skal informeres om deres innleggelse i sykehus vil heller ikke takke ja til deltakelse i studien og har i praksis anledning til å reservere seg mot at det sendes epikrise til fastlegen).
6) Hva med pasienter hvor dette var en engangsforeteelse, og som ønsker å legge det bak seg?
Dersom pasienter ikke ønsker å besvare spørsmål som omhandler selvmordstanker et halvt år etter en impulshandling er det tydeliggjort i følgebrevet som sendes i posten etter tre og seks måneder at det er anledning til å trekke seg på et hvilket som helst tidspunkt uten å måtte oppgi noen grunn.(Ved tilfeller av forgiftning hvor det er åpenbart at det er er et uhell eks.kullosforgiftning ved brann eller et rent ønske om rus vil ikke inkluderes i studien.)
7) Deltakelse for fastleger: Kompensasjon eller idealisme?
En av de sentrale utfordringene er å oppnå tilstrekkelig deltakelse fra fastlegene. Det har vært drøftet om det er aktuelt å gi en kompensasjon. Temaet har blitt diskutert med fastleger og etisk komite pr telefon. Legemiddelindustrien gir ofte honnorar til fastlegene. Ulempen med å gi slike honnorarer vil være at det underbygger en tendens hvor penger må til for at helsepersonell skal delta i kliniske forskningsprosjekter. I denne studien har fastlegene allerede et pålagt ansvar for å følge opp pasienter på sin liste. Deltakelse i studien vil gi fastlegene veiledning og skrftlig materiale. Betalingen for pasientkonsultasjonene dekkes av NAV og pasient.
8) Dersom pasienter utvikler alvorlig selvmordsatferd i oppfølgingsperioden?
Pasienter som måtte utvikle selvmordsfare vil bli vurdert av fastlegene ev. samarbeid med prosjektledelsen og/eller DPS, og de vil bli sikret ev. tettere oppfølging /innleggelse i psykiatrisk avdeling hvis det er nødvendig. Oppfølgingen blir således tettere enn det som er rutine. Hvis de som er randomisert til vanlig behandling skulle henvende seg til prosjektledelsen, vil det bli iverksatt nødvendige oppfølgingstiltak.
9) Har prosjektledelsen og prosjektdeltakerne tilstrekkelig kompetanse til å ivareta en sårbar gruppe pasienter?
Prosjektgruppens sammensetning er detaljert beskrevet i prosjektbeskrivelsen og det henvises dit.
Prosjektdeltakerne er de samme som vurderer og behandler denne pasientgruppen i det daglige kliniske og ansees å være istand til å iverksette adekvate tiltak for pasienter som deltar i studien.
Oppsummering:
Slik vi ser det er manglende oppfølging og delvis fraværende forskning rundt tiltak et av de største etiske dilemmaene innenfor selvmordsfeltet.

11. Informasjon og samtykke

Studien inkluderer personer med full samtykkekompetanse
- (•) Ja
- ( ) Nei

Studien inkluderer personer med redusert samtykkekompetanse
- ( ) Ja
- (•) Nei

Studien inkluderer personer med manglende samtykkekompetanse
- ( ) Ja
- (•) Nei

Samtykke skal ikke innhentes
- ( ) Ja
- (•) Nei

Hvis ja: begrunnelse

Ikke aktuelt.

Redegjør for hvordan forsøkspersonene/informantene rekrutteres og gis informasjon om prosjektet, dets formål, eventuelle risiki, rett til å avbryte o.a.

Deltakere rekrutteres gjennom skriftlig og muntlig informasjon av helsepersonale som arbeider med denne pasientgruppen til daglig.
Formål er beskrevet i informert samtykke.
Deltakerne blir skriftlig informert om anledning til å trekke seg uten å oppgi noen begrunnelse på at hvilket som helst tidspunkt.

12. Prosjektleder/prosjektmedarbeideres forhold til forsøkspersonene/informantene

Redegjør for prosjektlederens/prosjektmedarbeideres forhold til forsøkspersonene/informantene (f.eks lege/pasient, lærer/student, overordnet/underordnet).

Sykepleier-pasient: pasienter som er inkluderbare vil få invitasjon og muntlig informasjon av sykepleiere i avdelingen.
Lege- pasient: ved Ullevål US. er det i hovedsak psykiater som foretar tilsyn av pasienter, og vil fylle ut Suicide Intention Scale. Pasienter i intervensjonsgruppen følges opp av sin fastlege.
Sosionom-pasient: det kan bli aktuelt å involvere sosionom som foretar tilsyn av pasienter i avdelingen, ved at sosionom ev forespør og assisterer pasienten i utfylling av spørreskjema.
Psykolog - pasient: ved Aker og Lovisenberg gjennomfører psykologer tilsyn av pasienter. Disse vil bidra til å fylle ut Beck Suicide Intention scale etter samtale med pasienten.
Psykiatrisk sykepleier- pasient: det psykiatriske oppfølgingsteamet ved Diakonhjemmet som vurderer pasienter består av psykiatrisk sykepleier og psykiater.
Lege-lege: Fastleger som deltar i intervensjonsgruppen vil få tilbud om å kontakte psykiater for å få veiledning.

13. Økonomiske sider ved prosjektet

A) Oppdragsgiver/sponsor

Type sponsor (velg fra liste og skriv navn)

- ☑ Egen institusjon
- ☐ Legemiddelfirma
- ☐ Annet privat firma
- ☐ Pasientorganisasjon
- ☐ Forskningsinstitusjon
- ☐ Norges forskningsråd
- ☐ Annen offentlig myndighet
- ☐ Annet

Hvem finansierer størstedelen av prosjektet? Egen institusjon

Sponsor 1

Navn

Adresse

Kontaktperson

Telefon

E-post

Sponsor 2

Navn

Adresse

Kontaktperson

Telefon

E-post

Sponsor 3

Navn

Adresse

Kontaktperson

Telefon

E-post

B) Ytelser til forsker

Redegjør for økonomiske ytelser til prosjektleder og medarbeidere fra farmasøytisk industri eller utstyrsleverandører i forbindelse med planlegging og gjennomføring av prosjektet. Redegjør også for evt. interessekonflikter for prosjektleder.

ikke aktuelt.

C) Ytelser til deltakerne

Redegjør for eventuell honorering/kompensasjon for forsøkspersonene/informantene.

ikke aktuelt.

14. Risiko

Gjør rede for risiki, som f.eks smerter, ubehag, psykiske påkjenninger uhell,komplikasjoner, og tiltak for å minske/forebygge disse.

For at pasientene ikke skal føle seg avvist, vil inklusjonsprosedyren inndeles i to faser: 1. Under oppholdet i sykehusavdelingen får pasientene til begge grupper informasjon om deltakelse. Intervensjonen med oppfølging hos fastlegen er her ikke detaljert beskrevet for å redusere kontrollgruppens grad av evt. skuffelse og følelse av ny avvisning ved å ikke få dette tilbudet. Pasienter som er forhåsndsrandomiert til intervensjonsgruppen blir ikke invitert av prosjektkoordinator før fastlegen evt har takket ja til å delta. Begrunnelsen er at det ikke vil være riktig å evt. ødelegge et tillitsforhold til fastlegen ved å få vite at han ikke er interessert. (mange pasienter får behandling for andre lidelser av sin fastlege).

Redegjør for om metodene er klinisk etablert eller ikke. Hvis de er nye, hvordan har prosjektleder og medarbeiderne tilegnet seg klinisk erfaring med dem?

Metodene er ikke etablert som en klinisk rutine, men tidligere forskning tyder på at et synlig engasjement kan virke bra for pasienter som har avvisning som et av sine sentrale problemområder. Tidlig intervensjon og oppfølging har gjennom Bærumsmodellen i Norge vist lovende resultater. Samarbeid med fastlegen er vanlig, men ikke standardisert prosedyre ved de ulike sykehusene. Fastlegen utgjør en nøkkelrolle mht behandling og koordinering av pasientbehandling i spesialist- og primærhelsetjenesten.

På hvilket grunnlag er risiko vurdert (dyreforsøk, pilotstudie, klinisk erfaring, etc.)?

Prosjektansvarlig Øivind Ekeberg har gjennom flere år arbeidet med denne pasientgruppen, og kan derfor sies å ha relevant klinisk erfaring. Risiko er derfor vurdert på grunnlag av klinisk erfaring, og er ikke aktuelt. (Snarere tvert om)

Beskriv hvordan komplikasjoner, bivirkninger, uventede hendelser, nye toksiske funn etc. blir registrert.

Registreres sammen med pasientens kode.

15. Forsikring

Forsøkspersonene/ informantene er dekket av følgende forsikring ved eventuelle uhell, komplikasjoner	☑ Pasientskadeerstatningsordningen<br>☐ Produktansvarsloven<br>☐ Særskilt forsikring<br>☐ Forsikring ikke aktuelt (skal begrunnes)

Om forsikring ikke er aktuelt, gi begrunnelse.

16. Legemiddelutprøving (gjelder kun for prosjekter med legemiddelutprøving)

Vil det bli brukt placebo i studien?	○ Ja<br>○ Nei

Dersom svaret er ja, begrunnelse.

Vil pasienter bli tatt av velregulert behandling?	○ Ja<br>○ Nei

Dersom svaret er ja, begrunnelse.

Utførte og planlagte studier og hvor mange pasienter som er inkludert i tidligere faser av utprøvingen.

Dosevalg av studiepreparatet.

Begrunnelse for dosevalg.

Valg av sammenligningspreparat og dosering av dette i forhold til studiepreparatet.

Hvis sammenligningspreparatet er et annet enn standard behandling, må dette begrunnes særskilt.

EudraCT-nummer

17. Beredskap

Redegjør for beredskap og oppfølging dersom grunnlaget for studien endres underveis, så som ved økt risiko, uventede hendelser, nye og/eller mer alvorlige bivirkninger. Drøft evt. behovet for interimanalyse og mulige tiltak, så som bruk av stoppgruppe, endring av design, endret informasjon til pasienter etc.

Dersom pasienter som randomiseres til rutine behandling henvender seg til prosjektledelsen vil det iverksettes nødvendige oppfølgingstiltak.
Dersom det identifiseres behov for behandling i forbindelse med besvarelse av spørreskjema vil det iverksettes tiltak.
En interimanalyse vil være et metodisk problem fordi det kan bidra til at intervensjonen oppjusteres ved dårlige resultater.

18. Vurdering/godkjenning av andre instanser

Statens legemiddelverk
- ○ Er/vil bli søkt/meldt
- ○ Er vurdert/godkjent av
- ◉ Er ikke aktuelt

Datatilsynet/Personvernon
- ○ Er/vil bli søkt/meldt
- ◉ Er vurdert/godkjent av
- ○ Er ikke aktuelt

Helse- og omsorgsdepartementet
- ○ Er/vil bli søkt/meldt
- ○ Er vurdert/godkjent av
- ◉ Er ikke aktuelt

Sosial- og helsedirektoratet
- ○ Er/vil bli søkt/meldt
- ○ Er vurdert/godkjent av
- ◉ Er ikke aktuelt

Andre
- ◉ Er/vil bli søkt/meldt
- ○ Er vurdert/godkjent av
- ○ Er ikke aktuelt

Ved andre, hvem? Tillatelse fra sykehusene hvor det rekrutteres pasienter

19. Publisering og sluttrapport

Vil prosjektleder publisere eller gjøre allment tilgjengelig negative så vel som positive resultater, i henhold til Helsinkideklarasjonen?	(x) Ja<br>( ) Nei

Dersom svaret er nei, må dette begrunnes særskilt.

Er det publiseringsrestriksjoner i prosjektet, for eksempel fra sponsor?	( ) Ja<br>(x) Nei

Avslutningsår for prosjektet	2012

Underskrift

Sted:	Dato:

Underskrift:
