# Supplementary material for: Effect of Systematic Follow-Up by General Practitioners after Deliberate Self-Poisoning: A Randomised Controlled Trial
Source: PLoS One. 2015 Dec 2;10(12):e0143934. doi: 10.1371/journal.pone.0143934 (PMC4667913; doi:10.1371/journal.pone.0143934)
Supplement: S3 File — (PDF) [file pone.0143934.s004.pdf]

Project description

Systematic follow-up by general practitioners after self-poisoning: a randomised controlled clinical trail

Phd candidate: Tine K Grimholt Master of Clinical Nursing Science, Ullevaal University Hospital
Main supervisor: Øivind Ekeberg Prof. Dr.med Ullevaal University Hospital, Dep of Behavioural Sciences in medicine
Additional supervisor: Dag Jacobsen Prof. Dr. med Ullevaal University Hospital
Additional supervisor: Ole Rikard Haavet Associate Professor Dr.med UiO Institute of General Practice and Community Medicine

1.0 Introduction
2.0 Background

- 2.1 Terminology
- 2.2 Suicide
- 2.3 Suicide attempt
- 2.4 Prevalence of acute poisonings
- 2.5 Prognoses
- 2.6 Aftercare
- 2.7 Previous research

3.0 Primary Care, competence and attitudes
4.0 Objectives
5.0 Methodology / Design

- 5.1 The study part 1 RCT
- 5.2 Randomization and implementation
- 5.3 Intervention
- 5.4 Treatment as usual
- 5.5 Measures
- 5.6 Statistical procedures
- 5.7 Sample size
- 5.8 Operability
- 5.9 The study part 2 Cross sectional survey

6.0 Publications

- 6.1 Articles
- 6.2 International conferences

7.0 Ethical considerations
8.0 Organization and description of the research group
9.0 Conclusions
10.0 Appendix

- 10.1 References
- 10.2 Table of Instruments, background- and outcome variables and times of measurement
- 10.3 Trial flow chart

Abbreviations: RCT: Randomized Controlled Trial, GP: General Practitioner, DSH: Deliberate Self Harm, NPR: The Norwegian Patient Register

1.0 Introduction

Suicidal behaviour is a global public health problem. Suicide attempts are much more common than completed suicides. Rates of completed suicide and attempted suicide are positively correlated (Hawton 1998). Development of effective treatment strategies for patients following suicide attempt is an important element in suicide prevention and the randomised controlled trial (RCT) is widely accepted as the gold standard in evaluation of clinical treatments (Arensmann 2001). A large number of patients are hospitalized because of deliberate self-poisoning; many have a certain degree of suicidal intentions. Many later repeat the overdoses, commit suicide or die at an early age from other causes. A substantial number are suffering from social disadvantage, psychiatric and physical illness and misuse of drugs and alcohol. Today we are not aware of what kind of aftercare and follow up these people receive after discharge from hospital with an average stay app. 36 hours. Great varieties in the type and extent of after treatment have been noticed, but there is no systematic knowledge concerning this.

2.0 Background

2.1 Terminology

Suicide researchers have tried to find satisfactory terms for the range of suicidal behaviours. The most commonly used terms are: attempted suicide, deliberate self-harm, parasuicide, self-poisoning or self-injury and self-mutilation. The terms deliberate self-harm, self-poisoning and self-injury does not describe the suicide intent, only the behaviour. The term parasuicide as used in the World Health Organization/ European Study on parasuicide, include all methods and avoid describing intent rather than implying lack of intent. In the present study we use the term suicide attempt to describe patients who engage in self- poisoning with presence of suicidal intention. Intention varies from “cry for help” up to seriously planned suicide attempts. Drug related overdoses from e.g. GHB and Cocaine are excluded, and clinically these episodes are characterized as accidental poisonings.

2.2 Suicide

Worldwide there are almost one million suicides each year, a global mortality rate of 16 per 100,000, or one death every 40 seconds (www.WHO/ mental health/ supre). In Norway approximately 500 suicides are registered every year. The annual rate in 2006 was 7.3/100 00 for females and 15.8/ 100 000 for males (www.SSB.no).

2.3 Suicide attempt

Suicide attempts are more common than completed suicides, and rates of completed suicide and attempted suicide are positively related (Hawton 1998). Attempted suicide is a major risk factor for completion of suicide (Kerkhof 2000). Accurate national statistics from Norway are unavailable; however, Hjelmeland and co-workers found that in the county of Sør-Trøndelag in the period 1995–99, the suicide attempt rates were 130/100.000 for females and 90/100.000 for males. This local epidemiological study indicates figures with 10-15 more admitted suicide attempts in hospitals and outpatient clinics than the registered numbers of suicide. Literature also suggests that suicidal phenomena (suicide attempts, deliberate self-harm, and suicidal plans, threats and thoughts) are underreported (Retterstøl 2004).

2.4 Prevalence of acute poisonings

Kopjar and co workers found that rates for patients hospitalized after acute poisonings in Norway 2000- 02 for woman were 44 per 100 000 and 94 for men (Kopjar, Dieserud, Wiik 2005). A recent cohort study in Oslo (2003-04), 3025 episodes of acute poisoning were registered, and of these 947 episodes needed treatment in hospital. The incidence of hospitalized patients was 210 per 100 000 for woman and 190 for men.

2.5 Prognoses

The risk of repetition of self-harm and of later suicide is high (Hawton 2004). A ten year follow up study of all patients admitted to medical wards in Oslo after self poisoning found that 28 % of the men and 17 % of the woman had died. Median age at admittance was slightly above 30 years. (Ekeberg et.al,1994). After 20 year, follow up of the same material, 45 % of the men and 30 % of the woman were dead (Bjørnaas et.al 2008). The suicide risk was 36 times higher than expected in the first five year- and 23 in the last period. These findings underline the serious prognoses and the mortality can be compared to heart attack and several different types of cancer.

2.6 Aftercare

When the patients are discharged from somatic hospital the original crisis can be reactivated because the causing problems are still present. Repeated assessment of suicidal ideation is therefore recommended shortly after discharge. Out patient consultation with GP or psychiatric services within a week have been recommended (Nasjonale retningslinjer for forebygging av selvmord i psykisk helsevern). Assessment includes careful consideration of the patient’s intent and beliefs about the lethality of the method used. Presence of suicidal intention, seriousness of poisoning, precautions against being discovered, and psychiatric illness are indicators of high suicide risk. Management after self-harm includes forming a trusting relationship with the patient, jointly identifying problems, ensuring support is available in a crisis, and treating psychiatric illness vigorously (Skegg 2004).

2.7 Previous research

The need for clinical intervention studies are addressed and strongly recommended (Hawton et.al 2006). In Norway the Norwegian Directorate for Health and Social Affairs made an inquiry to the Norwegian Knowledge Centre for the Health Services (NOKC) to do a health technology assessment (HTA) of the effects of interventions by the psychiatric health services for the prevention of suicide. It was found that there is no evidence-based research from Norway, and a lack of methodological and well-designed international studies that fulfils the need for evidence based and clinical knowledge in treatment of patients after a suicide attempt. The conclusion is that there is a considerable need for research into the effects of different kinds of interventions offered suicidal patients (Mehlum et.al 2007). Repetition of DSH has been the sole outcome variable investigated in most studies. It is also recommended to investigate whether there is evidence of benefits with regard to outcomes like depression, hopelessness and suicidal ideation (Nordentoft 2007).

There are three main groups of suicide preventive methods:

Psychotherapy: this method has given some promising results, especially Dialectical Behavioural Therapy (DBT). In one study (Linehan 1991), there was a significantly lower rate of repetition of self-harm during the follow-up period in patients who received DBT.

Pharmacological therapy: there are few studies and therefore treatments advices have to be based on indirect evidence e.g.; antidepressants are effective in the treatment of depression and it could be anticipated that they also could prevent depression among suicidal patient and by this even suicide.

Other methods: this group comprises of different but often relatively uncomplicated methods, some have found promising results and will be shortly described: *Follow up and chains of care:* there are several models, but the most important and common element is the direct contact from health services shortly after an episode of hospitalisation following self harm. In

Norway the “Baerum model” showed decreased suicide attempt rates in the period 1995-1999 (Dieserud 2000). The problem however is that the study design cannot provide sufficient evidence, as there were no control group. *Emergency card:* in these models the patients is given a card with a telephone number to health professionals. Two studies found a trend towards less repetition of self-harm in the intervention group vs. control but the summary odds ratio of 0.45 (0.19 to 1.07) was not significant (Morgan 1993) Promising results have been found in studies of *telephone contact* (Vaiva et al., 2006) and *letter contact* were the suicide attempters received a post card after disharge from hospital (Motto & Bostrom, 2001; Carter et al., 2005).

3.0 Primary care, competence and attitudes

A high percentage of suicide victims have seen a primary care physician in the months before committing suicide. Thus, primary care physicians may play an important role in suicide prevention. Sudak and co-workers’ findings indicated a need for standardized curricular materials on suicide and depression. The findings suggest that experts could provide standardized curricula to primary care residencies in the recognition and management of suicide and depression. More robust training about these vital mental health concerns in primary care could reduce morbidity and mortality (Sudak 2007). A challenge in the aftercare is to assure continuity in the treatment. Cooperation between the levels of health care is necessary to create a good multidisciplinary climate. To overcome these challenges, it is important to address the need for increased competence and favourable attitudes to suicidal behaviour in health professionals (Mehlum 2007). Hawton and co-workers highlight the role of health professionals’ attitudes towards patients presenting with suicidal behaviour. Educational training in sensitivity and communication skills is important, as many have experienced negative attitudes. Especially important is this when the patients are vulnerable and afraid to seek help (Hawton 2008).
Studies of Norwegian physician’s attitudes and competence to treat patients with suicidal behaviour have not been performed.

4.0 Objectives

To examine the evidence for the effectiveness of a pragmatic and compound clinical intervention designed to reduce factors related to suicidal behaviour in adults admitted to hospital after self-poisoning.
More specifically the objectives are:

1. To test the hypothesis that follow up from general practitioners are more effective for patients after a suicide attempt than standard aftercare and a difference in suicidal ideation, depression, hopelessness, and further suicidal attempts.
2. To test the hypothesis that patients in the intervention group will have more contact with the general practitioner (better compliance) and be more satisfied with the follow up and treatment.
3. a)To study the attitudes to patients with suicidal behaviour among general practitioners and b) to test the hypothesis that there is a positive correlation between the attitudes of the general practitioners and the compliance and satisfaction of the patients.
4. To carry out a survey about attitudes and competence to patients with suicidal behaviour among GPs and physicians in medical and psychiatric wards

5.0 Design and methodology

5.1 The study part 1 RCT

Source of participants: patients admitted to the medical departments and emergency rooms following self-poisoning with suicidal intention in Oslo University Hospital Ullevål and Aker Diakonhjemmet and Lovisenberg (See flow chart)
Numbers: 200; 100 intervention group, 100 controls. Patients with GP who do not participate in the trail will be followed in a comparison group (n= Unknown).
Inclusion criteria: 1) age 18-75 years 2) no psychosis, mental retardation, or organic cognitive impairment; 3) not requiring psychiatric inpatient treatment: 4) ability to read and write Norwegian.

5.2 Randomization and implementation

*D*etermination of whether a patient will receive intervention or treatment as usual will be made by reference to a statistical series based on random sampling numbers. The numbers will be connected to the patients name before they consent. Concealment of the allocated intervention at the time of enrolment is especially important and therefore the numbers will be unknown for the ward staff in the enrolments of the participants. The assignment procedure will be performed in two steps. The informed consent will include information about whether they agree to receive a phone call from the project coordinator for participating in the intervention and to be followed up with their personal identification number in medical records. The co-ordinator will receive the randomization numbers when the enrolled are already connected to the patient's informed consent and filled in questionnaire with the same code. To avoid bias, it is necessary to separate the creation of the allocation sequence from assignment to the study groups. The allocation list will be stored at the secretary's office, and will therefore not be available to co-ordinator when performing in enrolment. This procedure will prevent any selection bias from the co-ordinator and enrolling participants. The numbers will be random and not predictable to assigners.

5.3 Intervention

The intervention in this clinical trail is compound and it is not possible to describe all variation and details in the general practitioners consultations or prospective treatment the patients will receive after discharge from the hospital. Nevertheless we will describe most of the treatment. The intervention has some systematic supplement elements:
Patients in the intervention group will get consultation with their GP as soon as possible- and maximum within two weeks after discharge. They receive follow up in a six-month period. This includes three consultations the first three months and two consultations the following three months. The GP can contact a psychiatrist for professional advice, supervision and discussion during the follow up period. The GP receive a written manual about assessment and treatment. It will contain suggestions about how they can ask the patient about symptoms, problems and psychiatric health status to provide adequate care. The manual is based on a document from a series of resources addressed to specific social and professional groups particularly relevant to the prevention of suicide. It has been prepared as part of SUPRE, the WHO worldwide initiative for the prevention of suicide.
The GP register and coordinate all kind of treatment the patient receives during the intervention period. This can be psychiatric hospitalization, psychiatric out patient clinic, further suicide attempts that requires medical treatment and other forms of health care. All contact with other facilities e.g. Social services, private psychologist / psychiatrist will also be registered.
The GPs in the intervention group answer a questionnaire about attitudes and competence to treat patients with suicidal behaviour at the time of inclusion and after six months.

5.4 Treatment as usual
It is recommended that studies in which treatment as usual is included should define precisely its nature. The treatment the patients receive will vary, but to provide a description

of this the data will be provided from the patients' self-report form, GP notes, verified from the hospitals records and the Norwegian Patient Register (NPR).

5.5 Measures
The instruments have tested for validity and reliability and have been translated into Norwegian (table appendix).

5.6 Statistical procedures

Means and proportions will firstly be compared by parametric and non-parametric comparisons. Multivariate analyses (ANOVA, multiple regression analyses) will be conducted. For scales, factor analyses, factor structure and inter item reliability analyses will be conducted. The analyses will be performed on an intention to treat basis. Statistical procedures will be conducted in cooperation with a statistician from the centre of clinical research. The statistical programme SPSS version 16.0 will be used.

5.7 Sample size
Beck Suicide Ideation Scale has shown prediction on suicide and suicide attempt (Guthrie et. al 2001). A five-point reduction on BSI will be clinically significant. A previous study found SD = 7,7. With alpha = 0,05 and Beta= 0,2 it is necessary to include 74 patients to each group. The study will include 200 patients to compensate for a possible drop out rate of up to 30%.

5.8 Operability

In a cohort study of all acute poisonings admitted to medical wards in Oslo 2003- 4, 748 patients were registered in the four participating hospitals. A new and slightly larger cohort were registered from our research group in 2008-09, preliminary results of intention show that approximately 70 % (n= 500) can be included in this trail. A previous study in 2005 using equal inclusion criteria like our study included 70% of the eligible patients. This is app.360 eligible patients from the four participating hospitals pr year.

5.9 The study part 2: Cross sectional survey

Design: A cross sectional survey will be carried out to describe and compare attitudes and competence to patients with suicidal behaviour between general practitioners and physicians in internal medicine and psychiatric wards.
Participants will be randomly recruited from address lists of all physicians in Norway in cooperation with the firm *Nomi.* They receive a postal questionnaire with a link to Quest back. The participants can choose to answer the questionnaire in an electronic or written form. An agreement has been made with Nomi to perform the collection of data and delivery of the complete data files.
Background variables: Gender, age, years of experience, speciality and education in treatment and assessment of patients with suicidal behaviour.
Instruments: *USP scale (Understanding of Suicidal Patients)* consisting of 17 items scored on a 5 point Likert scale (Samuelsson 1997). The form also contains questions about competence, empathy and involvement of suicidal patients compared to other patient groups (i.e. cancer, heart disease and opiate abusers).

6.0 Publications

The results from this project will be published in international and Norwegian peer-reviewed journals, international congresses and courses for clinicians in Norway. The trial is planned and will be reported in line with the CONSORT statement for correct reporting of RCTs. The

Vancouver guidelines for publishing will be followed. The authorship has partly been determined.

6.1 Articles

1) The effect of systematic follow-up of patients after a suicide attempt from general practitioners in Oslo: A randomised controlled trial
2) Attitudes and competence to suicidal patients among general practitioners and physicians in internal medicine and psychiatry in Norway
3) Attitudes and competence to somatic illness compared to suicidal behaviour
4) The effect of supervision and written guidelines on general practitioners attitudes and competence to treat suicidal patients
5) The role of the general practitioners in the follow up of patients after a suicide attempt

6.2 Planned presentations on international congresses

"Attitudes and competence to suicide attempt among general practitioners and physicians in medical and psychiatric wards in Norway"
13th European Symposium on Suicide and Suicide Behaviour, Rome, Italy International Association for Suicide Prevention 1-4 sept 2010

7.0 Ethical considerations

The Personvernombudet at Oslo University Hospital have approved the project and drafted the information letters and consents. The Regional Committee for Medical Research Etics (REK) South East Norway also approves the project. People with suicidal behaviours are sensitive and vulnerable. The ethical considerations have therefore been thoroughly evaluated in a close dialog with the ethical committee. One of the important aspects was precaution against patients who could develop serious suicidal ideation during the trail. Patients in the intervention group will be assessed and receive adequate treatment from their general practitioner in cooperation with the project leaders and/ or DPS to provide more intensive care or admission to psychiatric inpatient treatment. Patients in control- and comparison group will have the opportunity to contact the project leaders on a project telephone and given help to receive adequate treatment. The trial will be performed in line with principles for medical research described in the Helsinki declaration.

8.0 Organization and description of the research group

Cooperation with the Regional Centre of Violence, Traumatic Stress and Suicide Prevention East and Southern region (RVTS) is established. Local special advisors from the RVTS suicide prevention team will contribute to organization of recruitment and data collection: OUS Aker psychologists Ingvild Hurlimann-Lindseth and Diakonhjemmet hospital psychiatric nurse Astrid Berge Norheim. At Lovisenberg hospital we have established cooperation with Gudmund Nordby who is the chief physichian in the medical appartement. All the hospitals have participated and cooperated with the research group from Ullevål in projects involving acute poisonings since 1984 and latest in 2008.
The applicant finished the master programme of Clinical Nursing Science in 2007 and has been working in the Acute Medical Appartement in Ullevål since 2002. She will organize and coordinate the project.The survey of Norwegian physician's attitudes and competence is ready to be conducted in cooperation with Nomi and Questback. Invitations and information letters about the trial have been sent to all GPs in Oslo and based on the answers lists of the participating are ready.

At the Department of Behavioural Sciences in Medicine at the University of Oslo, there is a high competence within suicidiology and epidemiology. Professor dr. med Øivind Ekeberg is a specialist in psychiatry and a central part in national and international suicidology research for 25 years. Ekeberg also contributed to a suicide prevention programme in Norway. Post doc dr. med Erlend Hem finished a doctoral thesis on suicide among different health professionals in 2004 and was also main supervisor for the PhD candidates: Anne Marie Berg (2004) and Tom Sterud (2007). At the Department of Psychology, University of Oslo, Bergljot Gjelsvik is a research fellow and has contributed to the planning and instruments in the present study.
Professor. dr. med. Dag Jacobsen is the medical director at the Acute Medical Department and leader of the ABC centre (Atomic, Biological and Chemical) and the National Centre of clinical toxicology at Ullevål University Hospital. He has worked extensively with self-poisoning patients for 25 years.
We have established cooperation with the Institute of general practice and community medicine (IASAM) UiO. Dr. Med Ole Rikard Haavet is additional supervisor and will contribute to the theoretical foundation of general practice. He is a specialist in general- and community medicine. He is working clinically as general physician and associate professor at UiO.

9.0 Conclusions

The present study is original and can answer central issues within suicidology and the Norwegian health care system. Because of the relative high response rate from participants in Scandinavian studies, it is possible to obtain high data quality. The trial will be in the international frontline because of the addressed need for intervention studies to patients with suicidal behaviour. It is not likely to obtain sufficient number of patients to demonstrate a statistical significant reduction in suicide attempt rates because of the large numbers of patients (n= 1800) necessary to obtain sufficient power (Frankel Gunnel 1994). It is not rational to perform such large-scale studies before a more pragmatic trail with suicide related outcome variables has given significant results. Further focus should not only be targeted on mortality and repetition. Suicidal ideation, depression and feeling of hopelessness are serious conditions and demands seriously attention together with the elevated prevalence of alcohol- /drug misuse, physical and psychiatric illness and difficult socioeconomic situation in this large group of patients.

10.0 Appendix

10.1 References

Arensmann E., Towsend E., Hawton K., Bremner S., Feldman E., Goldney R.,Gunnel D et.al (2001) Psychsocial and pharmacological treatment of patients following deliberate self harm: the methodological issues involved in evaluating effectiveness *Suicide Life Threat Behav*. 2001 Summer;31 (2): 169-80

Bjørnaas MA., Jacobsen D., Haldorsen T., Ekeberg O. (2008) Mortality and causes of detah after hospital treated self poisionings in Oslo: a 20-year follow up *Clin Yoxicol* (Phila) Apr 17:1-8

Carter GL, Clover K, Whyte IM et al.(2005) Postcards from the EDge project: randomised controlled trial of an intervention using postcards to reduce repetition of hospital treated deliberate self poisoning. *BMJ;* 331;805–7.

Dieserud G. (2000) *Suicide attempt Unsolvable lives?* Dr. psychol.dissertation, University of Oslo Norway ISBN: 82-569-1592-7

Ekeberg Ø, Jacobsen D, Ellingsen Ø. (1994) Mortality and causes of death in a 10-year follow-up of patients treated for self-poisonings in Oslo. *Suicide Life Threat Behav* 1994; 24: 398–405.

Gould MS., Greenberg T., Velting DM., Shatter D. (2003) Youth risk and preventive interventions:a rewiew of the past ten years *J Am Acad Child Adolesc Psychiatry* Apr; 42 (4) :386-405

Gunnell D, Frankel S. (1994) Prevention of suicide: aspirations and evidence. *BMJ* 1994; 308: 1227–33.

Hawton K., Arensmann E., Towswnd E., Bremmner S., Feldman E., Goldney R., et.al (1998) Deliberate self harm : Systematic review of efficacy of psychosocial and pharmacologicaltreatments in preventing repetition *BMJ* Aug 15;317 (7156):441-7

Hawton K, Townsend E, Arensman E. et al (2006) Psychosocial and pharmacological treatments for deliberate self harm. *Cochrane Database of Systematic Reviews* 2
Hawton K. (2008) Plenary speaker at the 12th European Symposium on Suicide and Suicidal Behaviour, Glasgow Scotland 28th aug 2008

Hjelmeland H.(2004) *Suicidal behaviour in Norway.* I: Schmidtke A, Bille-Brahe U, De Leo D et al., red. Suicidal behaviour in Europe. Cambridge, Mass.: Hogrefe & Huber, 57–69.

Kerkhof AFJM (2000). Attempted suicide: Patterns and trends. *The international handbook of suicide and attempted suicide.* Chichester, UK: Wiley, 2000:49-64

Kopjar B, Dieserud G, Wiik J. Selvpåførte forgiftninger behandlet i sykehus. Tidsskr Nor Lægeforen 2005; 125: 1798–800.

Mehlum L. Dieserud G. Ekeberg Ø., Grøholt B., Mellesdal L., Walby F., Myhre K. (2007) Forebygging av selvmord, del 2. Rapport fra Kunnskapssenteret nr 04 –2007. ISBN 978-82-8121-146-9 ISSN 1890-1298

Morgan JA, Jones EM, Owen JH (1993) Secondary prevention of non-fatal deliberate self harm. The green card study. British Journal of Psychiatry 163:111-2

Motto JA., Bostrom AG. (2001) A randomized controlled trial of postcrisis suicide prevention. *Psychiatr Serv* 2001; 52: 828–33.

Nordentoft M. (2007) Prevention of suicide and attempted suicide in Denmark. Epidemiological studies of suicide and interventions in selected risk groups *Dan Med Bull* Nov;54 (4):306-69

Nasjonale retningslinjer for forebygging av selvmord i psykisk helsevern. Oslo: Sosial- og helsedirektoratet, 2008. www.shdir.no/vp/multimedia/archive/00036/IS1511_Selvmord_kor_36439a.pdf(14.11.2008).

Retterstøl N., Ekeberg Ø., Mehlum L. (2004) *Selvmord: et personlig og samfunnsmessig problem*, Gyldendal Akademisk. Oslo

Skegg K. (2005) Self Harm *Lancet* Oct 22-28;366 (9495) : 1471-83

Sudak D., Roy A., Sudak H., Lipschitz A., Maltsberger J., Hendin H. (2007) Defencies in suicide trainingin primary care specialities: a survey of training directors *Acad Psychiatry* Sep-Oct; 31(5): 345-9

Vaiva G., Ducrocq F., Meyer P., et al. (2006) Effect of telephone contact on further suicide attempts in patients discharged from an emergency department: randomised controlled study. *BMJ 2006; 332: 1241–4.*

(www.SSB.no) and (www.WHO mental health /suicide prevention /SUPRE)

10.2
TABLE OF BACKGROUND- / OUTCOME VARIABLES AND MEASUREMENT TIMES

Patients included in trial Intervention and Control groups (n=200)	Sociodemographic Background variables Registrations at assignment	Measures T1 Hospital (self report questionnaire)	Measures T2 3 months (self report questionnaire)	Measures T3 6 months (self report questionnaire)	Registrations (From GP, NPR, ambulance,hospital, outpatient clinics and self report)
	Age, Gender, psychiatric diagnoses (ICD-10), Suicide Intention *(Beck Suicide Intention Scale SIS),* marital status, occupation, Previous suicide attempt, previous treatment, somatic disease	Beck Scale for Suicide Ideation (BSI), Beck Hopelessness Scale (BHI), Beck Depression Inventory (BDI)	Beck Scale for Suicide Ideation (BSI), Beck Hopelessness Scale (BHI), Beck Depression Inventory (BDI) Changes in marital status	Beck Scale for Suicide Ideation (BSI), Beck Hopelessness Scale (BHI), Beck Depression Inventory (BDI) Changes in marital status Satisfaction with GP (EUROPEP)	Consultations, telephone and letter contact with GP Compliance with treatment plans, treatment in other health care services, Repetition of self-harm included methods, completed suicide
GPs included in intervention group (n=100)	Background variables (Registered from self report)	Immediately after patient assignment (Questionnaire)	After six months end of intervention (Questionnire)		
	Age, gender, years of experience, specialty, received education> 5 years	USP scale (Understanding of suicidal patients) Perceived need for competence	USP scale (Understanding of suicidal patients) Perceived need for competence		
GPs (n=200), Physicians in medical wards (n=200) and Physicians in psychiatric wards (n=200)	Age, gender, years of experience, specialty, received education> 5 years	USP scale (Understanding of suicidal patients) Perceived need for competence			

FLOW CHART

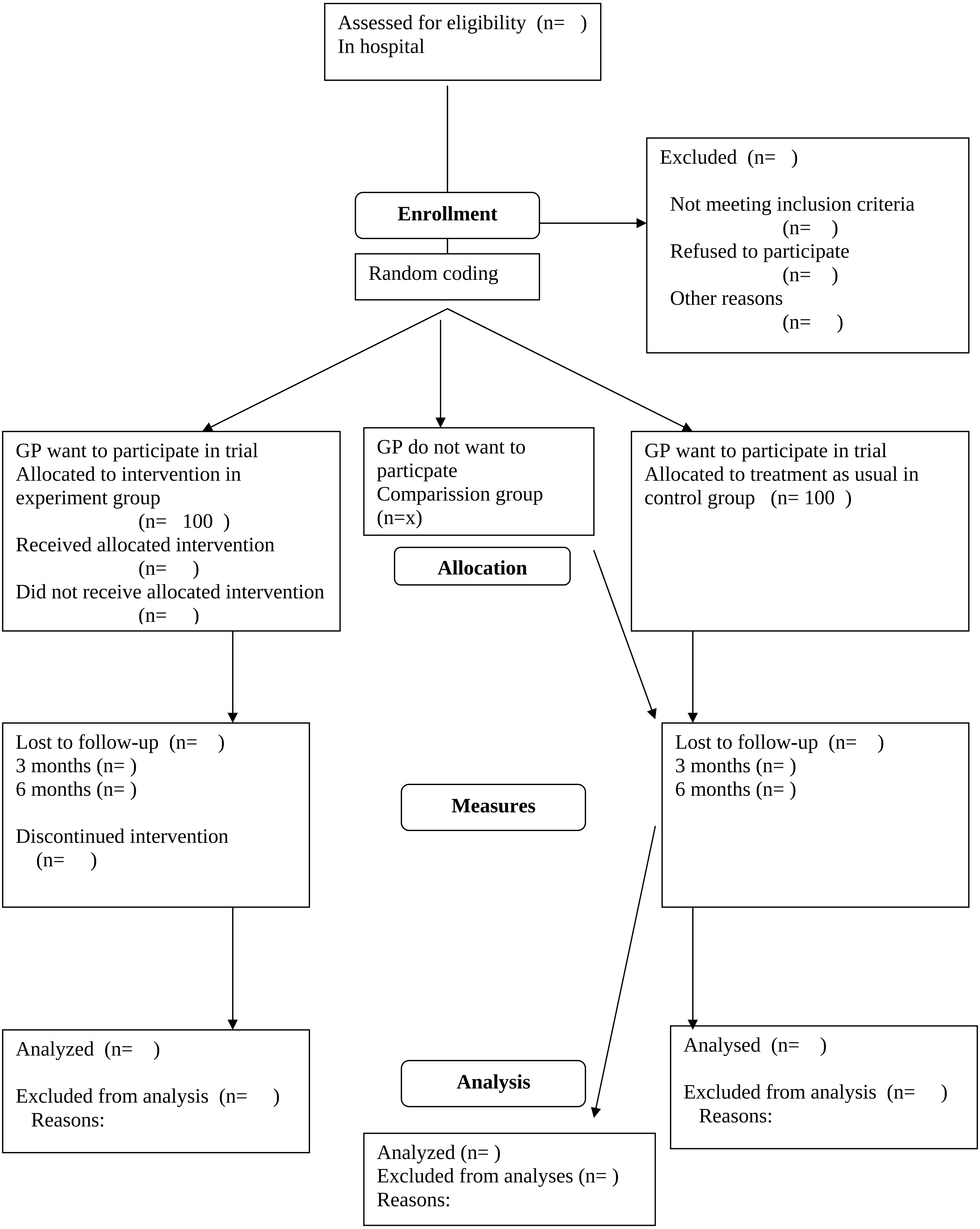
Assessed for eligibility (n=)
In hospital
Enrollment
Excluded (n=)
Not meeting inclusion criteria (n=)
Refused to participate (n=)
Other reasons (n=)
Random coding
GP want to participate in trial
Allocated to intervention in experiment group (n= 100)
Received allocated intervention (n=)
Did not receive allocated intervention (n=)
GP do not want to particpate
Comparission group
(n=x)
GP want to participate in trial
Allocated to treatment as usual in control group (n= 100)
Allocation
Lost to follow-up (n=)
3 months (n=)
6 months (n=)
Discontinued intervention (n=)
Measures
Lost to follow-up (n=)
3 months (n=)
6 months (n=)
Analyzed (n=)
Excluded from analysis (n=)
Reasons:
Analysis
Analysed (n=)
Excluded from analysis (n=)
Reasons:
Analyzed (n=)
Excluded from analyses (n=)
Reasons:
